# Rab5-dependent autophagosome closure by ESCRT

**DOI:** 10.1083/jcb.201811173

**Published:** 2019-04-22

**Authors:** Fan Zhou, Zulin Wu, Mengzhu Zhao, Rakhilya Murtazina, Juan Cai, Ao Zhang, Rui Li, Dan Sun, Wenjing Li, Lei Zhao, Qunli Li, Jing Zhu, Xiaoxia Cong, Yiting Zhou, Zhiping Xie, Valeriya Gyurkovska, Liuju Li, Xiaoshuai Huang, Yanhong Xue, Liangyi Chen, Hui Xu, Haiqian Xu, Yongheng Liang, Nava Segev

**Affiliations:** 1 College of Life Sciences, Key Laboratory of Agricultural Environmental Microbiology of Ministry of Agriculture and Rural Affairs, Nanjing Agricultural University, Nanjing, China; 2 Department of Biochemistry and Molecular Genetics, College of Medicine, University of Illinois at Chicago, Chicago, IL; 3 State Key Laboratory of Microbial Metabolism, School of Life Sciences and Technology, Shanghai Jiao Tong University, Shanghai, China; 4 Department of Biochemistry and Molecular Biology, Dr. Li Dak Sam and Yap Yio Chin Center for Stem Cell and Regenerative Medicine, Zhejiang University School of Medicine, Hangzhou, China; 5 State Key Laboratory of Membrane Biology, Beijing Key Laboratory of Cardiometabolic Molecular Medicine, Institute of Molecular Medicine, Peking University, Beijing, China; 6 The National Laboratory of Biomacromolecules, Chinese Academy of Sciences Center for Excellence in Biomacromolecules, Institute of Biophysics, Chinese Academy of Sciences, Beijing, China

## Abstract

In the conserved autophagy pathway, autophagosomes (APs) engulf cellular components and deliver them to the lysosome for degradation. Before fusing with the lysosome, APs have to close via an unknown mechanism. We have previously shown that the endocytic Rab5-GTPase regulates AP closure. Therefore, we asked whether ESCRT, which catalyzes scission of vesicles into late endosomes, mediates the topologically similar process of AP sealing. Here, we show that depletion of representative subunits from all ESCRT complexes causes late autophagy defects and accumulation of APs. Focusing on two subunits, we show that Snf7 and the Vps4 ATPase localize to APs and their depletion results in accumulation of open APs. Moreover, Snf7 and Vps4 proteins complement their corresponding mutant defects in vivo and in vitro. Finally, a Rab5-controlled Atg17–Snf7 interaction is important for Snf7 localization to APs. Thus, we unravel a mechanism in which a Rab5-dependent Atg17–Snf7 interaction leads to recruitment of ESCRT to open APs where ESCRT catalyzes AP closure.

## Abstract (precis)

Zhou et al. identify the mechanism of autophagosome (AP) closure. They show that Rab5 GTPase regulates an interaction between the ESCRT subunit Snf7 and Atg17 to bring ESCRT to APs where it catalyzes AP closure. These findings highlight the convergence of the endocytic and autophagic pathways at this step.

## Introduction

Autophagy is a recycling pathway that shuttles surplus and damaged cellular components for degradation in the lysosome under normal and stress conditions. Defects in this pathway are associated with a myriad of human diseases (Rubinsztein et al., 2012; Choi et al., 2013). Whereas macro-autophagy is mediated by autophagy-related proteins (Atgs) and double-membrane autophagosomes (APs) that fuse with the lysosome (Weidberg et al., 2011; Feng et al., 2014), in micro-autophagy, cargo is directly engulfed by the lysosomal membrane (Oku and Sakai, 2018). Most of the research on macro-autophagy has centered on its inception, including signaling pathways that induce it, their effect on Atgs, and the assembly of the preautophagosomal structure by Atgs (Ohsumi, 2014; Mercer et al., 2018). Understanding of early autophagy steps originated from studies in yeast, and later the machinery and mechanisms were confirmed to be highly conserved (Nakatogawa et al., 2009).

In comparison to early steps, much less is known about the late steps of the macro-autophagy, especially about AP maturation. During maturation, the double membrane seals to form closed APs, which then lose most of the Atgs. Mature APs can fuse with the lysosomal membrane and release single-membrane autophagic bodies (ABs) into the lysosome (Klionsky and Eskelinen, 2014). Fusion of APs and late endosomes with the lysosome is regulated and mediated by a similar set of factors that include the Ypt7 GTPase module and the cellular fusion machinery of tethers and SNAREs (Numrich and Ungermann, 2014). We have recently shown that the endocytic and autophagic pathways intersect in a step earlier than fusion with the lysosome, which is regulated by the Rab5-GTPase module (Chen et al., 2014). Moreover, we showed that the Rab5 module is important for AP closure (Zhou et al., 2017). These findings raised the possibility that endocytic machinery that functions downstream of Rab5 is involved in AP sealing. However, AP sealing cannot be catalyzed by the cellular membrane-fusion machinery (e.g., tethers and SNAREs), which catalyzes “pointed” fusion between two membranes. This is because AP sealing requires membrane fusion along a “rim of a cup,” which topologically resembles membrane scission. The ESCRT complex can catalyze such a process (Hurley and Hanson, 2010).

ESCRT is a set of conserved complexes—0, I, II, and III—and a number of accessory proteins (e.g., the Vps4 ATPase). ESCRT was originally identified as the machinery that mediates scission of intraluminal vesicles (ILVs) into late endosomes, also termed multivesicular bodies (MVBs) in yeast. The cytosolic ESCRT complexes function in concert to bind ubiquitinated proteins on endosomal membranes, cluster them to specific membrane domains, constrict these domains, and catalyze pinching off vesicles containing these domains into the lumen of the endosome (Henne et al., 2013). More recently, ESCRT was shown to mediate topologically similar membrane constriction and abscission in other cellular processes, e.g., plasma membrane (PM) abscission during cytokinesis and PM repair, exosome and microvesicle shedding, and viral release from the PM (Alonso Y Adell et al., 2016; Christ et al., 2017). In endocytosis, ESCRT functions after Rab5 GTPase (Russell et al., 2012).

Based on our finding that the autophagy and endocytosis merge at a Rab5-regulated step (Chen et al., 2014; Zhou et al., 2017), that in endocytosis Rab5 functions before ESCRT (Russell et al., 2012), and that the topological similarity between membrane scission of MVB vesicles and AP sealing (Hurley and Hanson, 2010), we speculated that ESCRT mediates AP sealing. However, currently the involvement of ESCRT in autophagy has two problems. First, there is conflicting evidence regarding the role of ESCRT in macro-autophagy in general and specifically in which step of this pathway (Nara et al., 2002; Filimonenko et al., 2007; Rusten et al., 2007; Sahu et al., 2011; Djeddi et al., 2012; Liu et al., 2015; Müller et al., 2015; Mukherjee et al., 2016; Hammerling et al., 2017; Oku et al., 2017; Lefebvre et al., 2018). Second, establishing a direct role for ESCRT in autophagy using traditional assays is challenging. This is because traditional assays mostly assess processing of autophagy cargo by lysosomal proteases, and ESCRT affects delivery of such proteases to the lysosome (Müller et al., 2015) and thereby could affect autophagic cargo processing indirectly.

Here, we establish that in yeast the ESCRT machinery plays a direct role in autophagy and catalyzes AP closure. Specifically, single- and double-gene knockouts together with genetic and biochemical complementation analyses indicate that cells depleted for the ESCRT III subunit Snf7 or the Vps4 ATPase exhibit autophagy defects and accumulate unsealed APs decorated with Atgs. Moreover, we show that the Rab5 GTPase Vps21 controls the localization of the ESCRT III subunit Snf7 and the Vps4 ATPase to APs and the interaction of Snf7 with Atg17. While the established role of Atg17 is in AP formation during stress-induced autophagy (Davies et al., 2015), it stays on APs until their maturation and was recently proposed to have a second role in a late step of autophagy (Liu et al., 2016). Finally, we show that the Rab5–dependent Atg17–Snf7 interaction is required for autophagy. Thus, we delineate a mechanism by which Rab5 GTPase controls ESCRT recruitment to unsealed APs to catalyze their closure.

## Results

### ESCRT mutants are defective in autophagy and accumulate AP clusters

In the endocytic pathway, the Rab5 GTPase and ESCRT play successive roles (Russell et al., 2012; Schuh and Audhya, 2014). Because we discovered that Vps21, a Rab5 GTPase, plays a role in autophagy in yeast cells (Chen et al., 2014), we decided to determine whether the ESCRT machinery also plays a role in this process. Currently, the evidence regarding a role for ESCRT in autophagy is inconclusive (Hurley and Hanson, 2010). Therefore, we started by determining whether ESCRT mutants are defective in stress-induced autophagy in yeast and, if defective, in which step.

Yeast cells deleted individually for each of the ESCRT subunits were tested for their ability to process two autophagy cargos upon starvation: Ape1 and GFP-Atg8. Upon nitrogen starvation, >90% of these two cargos reach the vacuole and get degraded in WT cells, whereas cells defective in autophagy accumulate undegraded proteins. The majority of the ESCRT mutants tested (13 of 20) exhibited partial defects in autophagy-cargo processing, similar for the two substrates (∼40–50% inhibition; Fig. S1). ESCRT mutants were also tested for GFP-Atg8 accumulation using live-cell fluorescence microscopy. In WT cells, GFP-Atg8 is delivered to the vacuole (marked by FM4-64) upon starvation. In contrast, all ESCRT mutants that exhibited processing defects of autophagy cargo (above), accumulated GFP-Atg8 in crescent-like structures next to their vacuoles (in ∼30–50% of the cells; Fig. S2), similar to those accumulated in mutant cells depleted of the Rab5 GTPase-module components (Chen et al., 2014). In *vps21Δ* mutant cells, we showed that these GFP-Atg8 structures represent AP clusters and below we show that this is also true for ESCRT mutants. These results show that subunits representative of each of the ESCRT complexes, 0–III, and the Vps4 ATPase play a role in stress-induced autophagy in yeast and suggest that, when depleted, the block is in a late step of autophagy, after AP formation.

**Figure 1.**
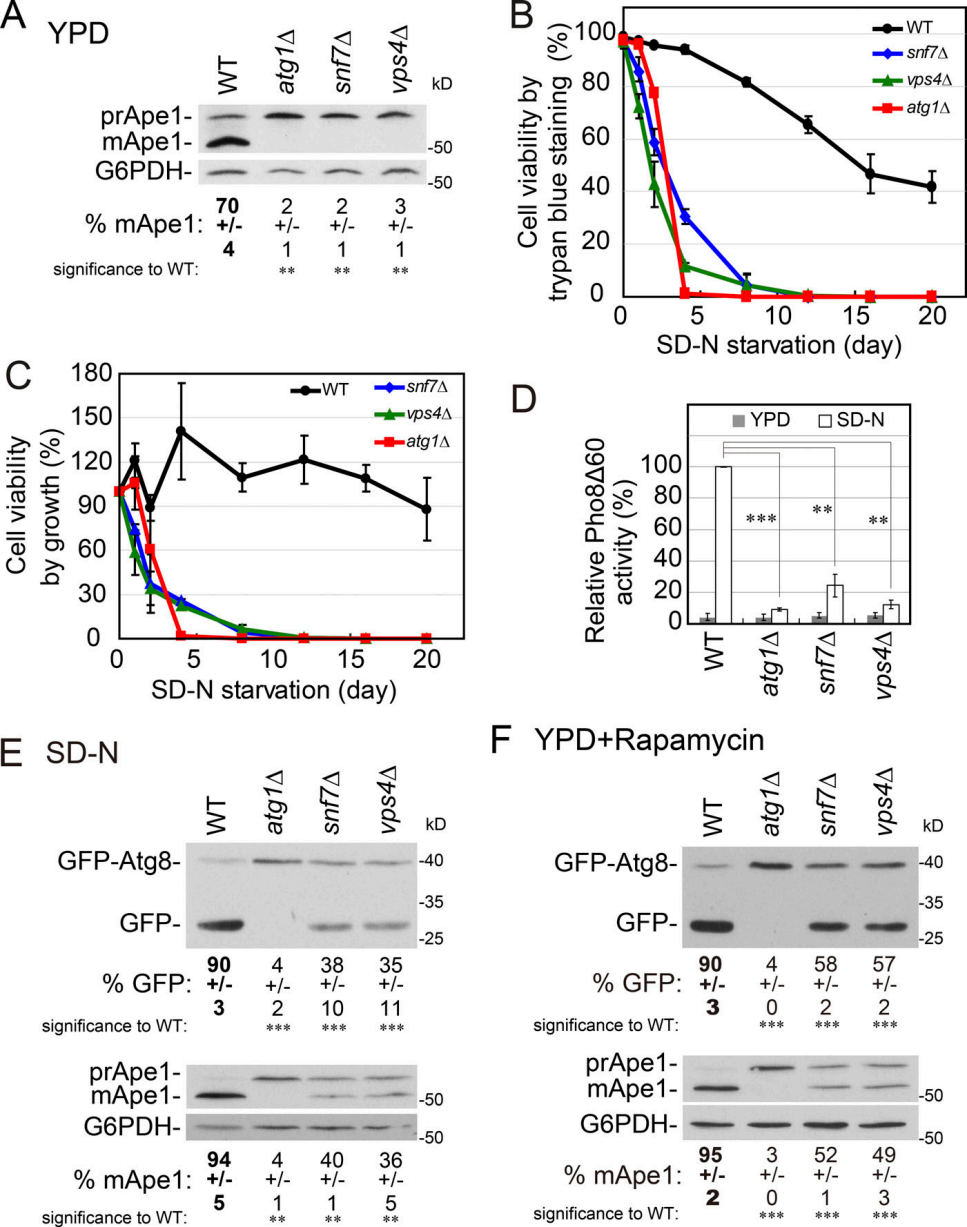
***snf7Δ*and *vps4Δ* ESCRT mutant cells exhibit selective and general autophagy defects. (A)** ESCRT mutants exhibit a complete block in Ape1 processing during normal growth. WT and mutant cells were grown in rich medium (YPD) to mid-log phase. Processing of pre-Ape1 (prApe1) to mature Ape1 (mApe1) was determined in cell lysates using immunoblot analysis with anti-Ape1; percentage of processed cargo in each lane is shown under the blot. **(B and C)** ESCRT mutants exhibit a severe loss of viability under nitrogen starvation. WT and mutant cells grown in YPD to mid-log phase were shifted to SD-N. Cell viability, shown as percentage of cell viability at day zero, was tested by trypan blue staining (B) and ability to form colonies on YPD plates (C) at time zero and after the indicated number of days in SD-N. **(D)** ESCRT mutant cells are defective in general autophagy measured by the Pho8Δ60 alkaline phosphatase assay. Alkaline phosphatase activity was determined in lysates of WT and mutant cells grown in YDP or SD-N (4 h). **(E)** ESCRT mutants exhibit a partial defect in GFP-Atg8 (top) and Ape1 (bottom) processing under starvation. WT and mutant cells expressing GFP-Atg8 were grown in YPD and shifted to starvation medium (SD-N for 2 h). Processing was determined in cell lysates by immunoblot analysis using anti-GFP and anti-Ape1 antibodies, respectively; percentage of processed cargo in each lane (GFP and mApe1) is shown under the blot. **(F)** ESCRT mutants are partially defective in GFP-Atg8 and Ape1 processing during rapamycin-induced autophagy. WT and mutant cells were grown to mid-log phase in YPD, and autophagy was induced by the addition of rapamycin (10 ng/ml for 4 h). GFP-Atg8 (top) and Ape1 (bottom) processing was determined and presented as in E. Error bars and +/− represent SD. Results represent three independent experiments. **P < 0.01; ***P < 0.001.

We focused on two ESCRT subunits that function in late steps of membrane scission in MVBs, the ESCRT III subunit Snf7 and the Vps4 ATPase (Hurley and Hanson, 2010). Depletion of Snf7 or Vps4 resulted in defects of ∼50% in both cargo processing and GFP-Atg8 cluster accumulation under starvation (Fig. S1 and Fig. S2). A more thorough analysis of selective and general autophagy phenotypes was performed for *snf7Δ* and *vps4Δ* mutant cells in comparison to *atg1Δ* mutant cells, which are completely defective in all types of macro-autophagy (Nakatogawa et al., 2009; Feng et al., 2014). First, a constitutive selective autophagy process termed cytosol-to-vacuole transport (CVT) was followed under normal growth conditions (rich medium) using Ape1 as a cargo. Both *snf7Δ* and *vps4Δ* mutant cells, like *atg1Δ*, are completely defective in CVT processing (Fig. 1 A). Second, the ability of cells to survive when starved for nitrogen was determined using two methods: vital staining and ability to form colonies. In both assays, *snf7Δ* and *vps4Δ* mutant cells exhibited severe viability defects similar to those of *atg1Δ* (Fig. 1, B and C). Third, both *snf7Δ* and *vps4Δ* mutant cells exhibited severe starvation-induced autophagy defects using a quantitative alkaline phosphatase (Pho8Δ60) assay (Fig. 1 D). Last, the processing of two cargos, Ape1 and GFP-Atg8, was followed upon induction of general autophagy by starvation (nitrogen-starvation medium [SD-N]) or addition of rapamycin. Under both conditions, *snf7Δ* and *vps4Δ* mutant cells exhibited partial defects of cargo processing (Fig. 1, E and F). All the autophagy defects of *snf7Δ* and *vps4Δ* mutant cells, viability and cargo processing, can be complemented by a functional cognate protein expressed from a plasmid (Fig. S3, A–D). Thus, using different assays and cargos, we established that in yeast the ESCRT mutants *snf7Δ* and *vps4Δ* exhibit complete autophagy defects in some assays and partial defects of starvation-induced cargo processing.

The next question we addressed was in which autophagy step these ESCRT mutant cells are defective. Upon induction of autophagy by starvation or rapamycin, GFP-Atg8 crescent-like structures were observed in the majority of *snf7Δ* and *vps4Δ* mutant cells, but not in WT or *atg1Δ* cells (Fig. S3, E and F). This phenotype of *snf7Δ* and *vps4Δ* mutant cells can be complemented by the cognate WT protein expressed from a plasmid (Fig. S3 G). The following experiments show that the Atg8 crescent-like structures that accumulate in *snf7Δ* and *vps4Δ* mutant cells represent AP clusters, similar to those that accumulate in *vps21Δ* mutant cells (Chen et al., 2014).

First, time-lapse microscopy shows that the GFP-Atg8 crescent-like structures that accumulate in *vps4Δ* mutant cells, as we previously showed for *vps21Δ* mutant cells, are composed of multiple Atg8 dots that move independently (Videos 1 and 2). Hereafter, these structures will be termed Atg8 clusters.

**Figure 2.**
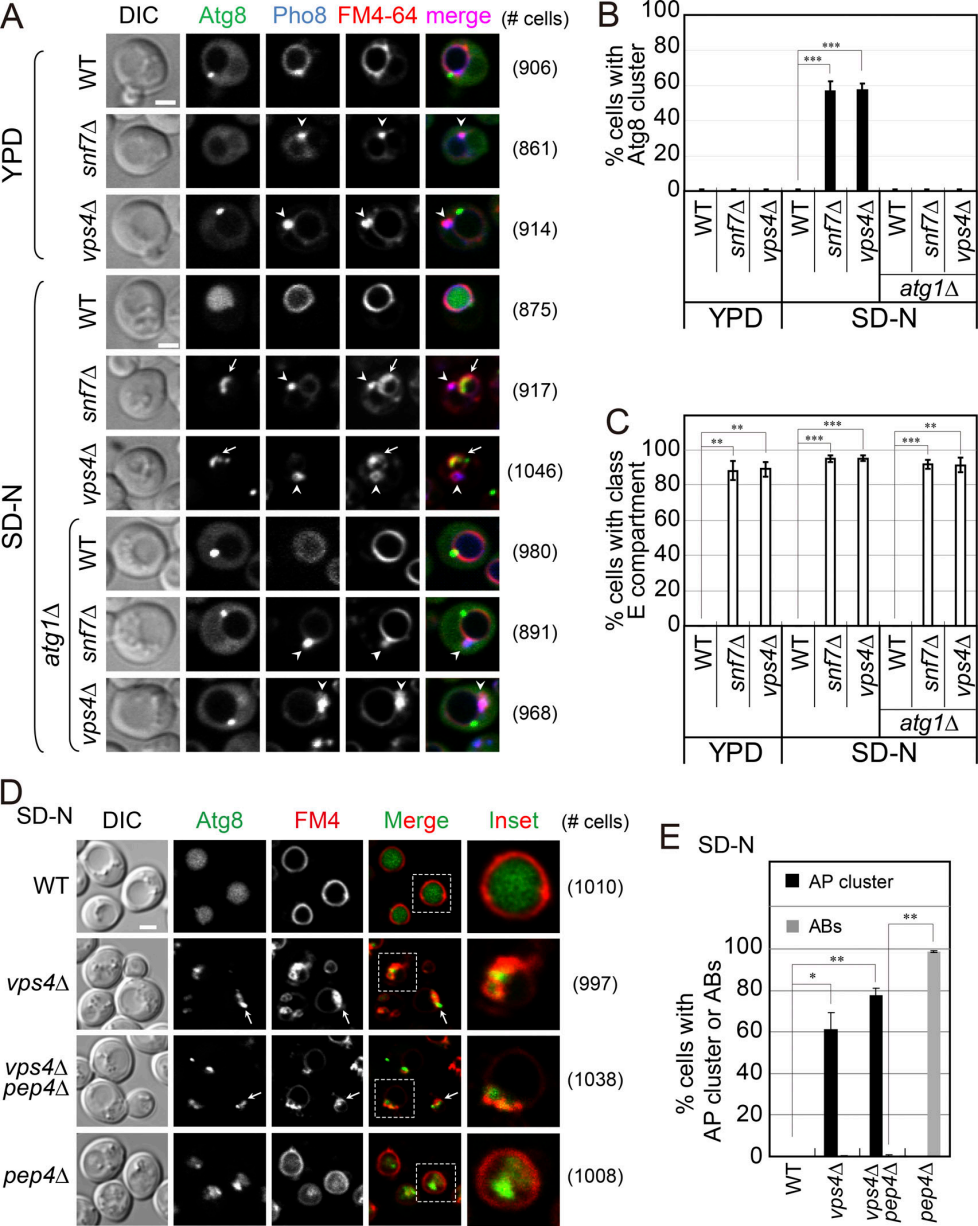
***snf7Δ*and *vps4Δ* ESCRT mutant cells accumulate GFP-Atg8 clusters under nitrogen starvation. (A–C)** Accumulation of Atg8 clusters and class E compartment in *snf7Δ* and *vps4Δ* single-mutant cells and in combination with the *atg1Δ* mutation. **(A)** Cells expressing GFP-Atg8 and the class E compartment marker 3×tagBFP-Pho8 were grown in YPD (top) and shifted to SD-N (bottom) as in Fig. 1 E. FM4-64 was used to label the vacuolar membrane before cells were visualized by live-cell microscopy (right, number of cells used for quantification in B and C). Arrowheads point to class E compartments; arrows indicate GFP-Atg8 clusters. Scale bar, 2 µm. **(B)** Atg8 clusters accumulate in *snf7Δ* and *vps4Δ* single-mutant cells only under starvation and not when combined with the *atg1Δ* mutation. Results from A were quantified and the bar graph shows percentage of cells with an Atg8 cluster. **(C)** Class E compartment accumulates in *snf7Δ* and *vps4Δ* single-mutant cells regardless of the growth conditions and the *atg1Δ* mutation. Results from A were quantified and percentage of cells with class E compartment is shown. **(D and E)** Epistasis analysis of GFP-Atg8 accumulation in single and double *vps4Δ* and *pep4Δ* mutant cells under nitrogen starvation. **(D)** Cells expressing GFP-Atg8 were grown in SD-N and FM4-64 was used to label the vacuolar membrane before visualization by live-cell microscopy (right, number of cells used for quantification in E). Arrows point to GFP-Atg8 clusters. Scale bar, 2 µm. **(E)** Whereas WT and *pep4Δ* mutant cells accumulate GFP-Atg8 inside the vacuole, *vps4Δ* and *vps4Δ pep4Δ* mutant cells accumulate GFP-Atg8 clusters outside the vacuole. Results from D were quantified, and percentage of cells with an Atg8 (AP) cluster outside the vacuole (black bars) or as ABs inside the vacuole (gray bar) are shown. In B–D: >800 cells per strain were counted; columns represent mean, error bars represent SD. Results in this figure represent three independent experiments. *P < 0.05; **P < 0.01; ***P < 0.001.

Second, ESCRT mutants accumulate an aberrant endosomal compartment termed class E, and we wanted to differentiate it from GFP-Atg8–marked APs. BFP-Pho8 (vacuolar alkaline phosphatase) and Cps1-NeonGreen (Cps1-NG; carboxy-peptidase S) were used as markers for the class E compartment. In WT cells, BFP-Pho8 localizes to the vacuolar membrane (colocalizes with FM4-64), whereas Cps1-NG is delivered to the vacuolar lumen; both are delivered to the vacuole via MVBs (Coonrod and Stevens, 2010; Russell et al., 2012). In *snf7Δ* and *vps4Δ* ESCRT mutant cells, class E compartment should accumulate regardless of the growth conditions, while AP clusters should accumulate only upon induction of autophagy. Indeed, *snf7Δ* and *vps4Δ* (but not *vps21Δ*) accumulate class E compartment (marked with either Pho8 or Cps1) when grown in rich medium or under starvation (SD-N). In contrast, Atg8 clusters accumulate in *snf7Δ*, *vps4Δ*, and *vps21Δ* mutant cells only under starvation, and class E compartment and GFP-Atg8 clusters do not overlap (Fig. 2, A–C; and Fig. S4, A and B). As expected, both phenotypes of *snf7Δ* and *vps4Δ* mutant cells, class E compartment accumulation, and Atg8 cluster formation can be complemented by the cognate protein expressed from a plasmid (Fig. S4, D and E). These results establish that, under starvation, in addition to the class E compartment, *snf7Δ* and *vps4Δ* mutant cells accumulate separate Atg8 clusters.

Double-mutant analyses were used to establish that the ESCRT machinery components function in the macro-autophagy pathway between Atg1 and Pep4. While deletion of *ATG1* in ESCRT mutant cells should prevent accumulation of AP clusters because AP formation is completely blocked in *atg1Δ* mutant cells, it should not have an effect on the accumulation of the class E compartment. Importantly, there is no Atg8-cluster accumulation in *atg1Δ snf7Δ* and *atg1Δ vps4Δ* double-mutant cells, while class E compartment is accumulated in the double-mutant cells (Fig. 2, A-C). These results support the idea that ESCRT mutants are defective in a late macro-autophagy step, after Atg1-mediated AP formation.

Mutant cells defective in vacuolar proteases (e.g., Pep4) accumulate ABs inside their vacuoles (Torggler et al., 2017). If in ESCRT mutant cells Atg8 clusters represent APs that did not fuse with the vacuole, the ESCRT mutant phenotype should mask that of the *pep4Δ* mutation. Using live-cell fluorescence microscopy, we show that whereas ABs marked with GFP-Atg8 accumulated inside the vacuoles of *pep4Δ* mutant cells (vacuolar membrane was marked with FM4-64), they did not accumulate inside the vacuoles of *vps4Δ pep4Δ* double-mutant cells. Instead, the double-mutant cells accumulated Atg8 clusters outside the vacuole, similar to those seen in the single *vps4Δ* mutant cells (Fig. 2, D and E). Thus, ESCRT mutants are defective in a step before delivery of autophagy cargo to the vacuole.

**Figure 3.**
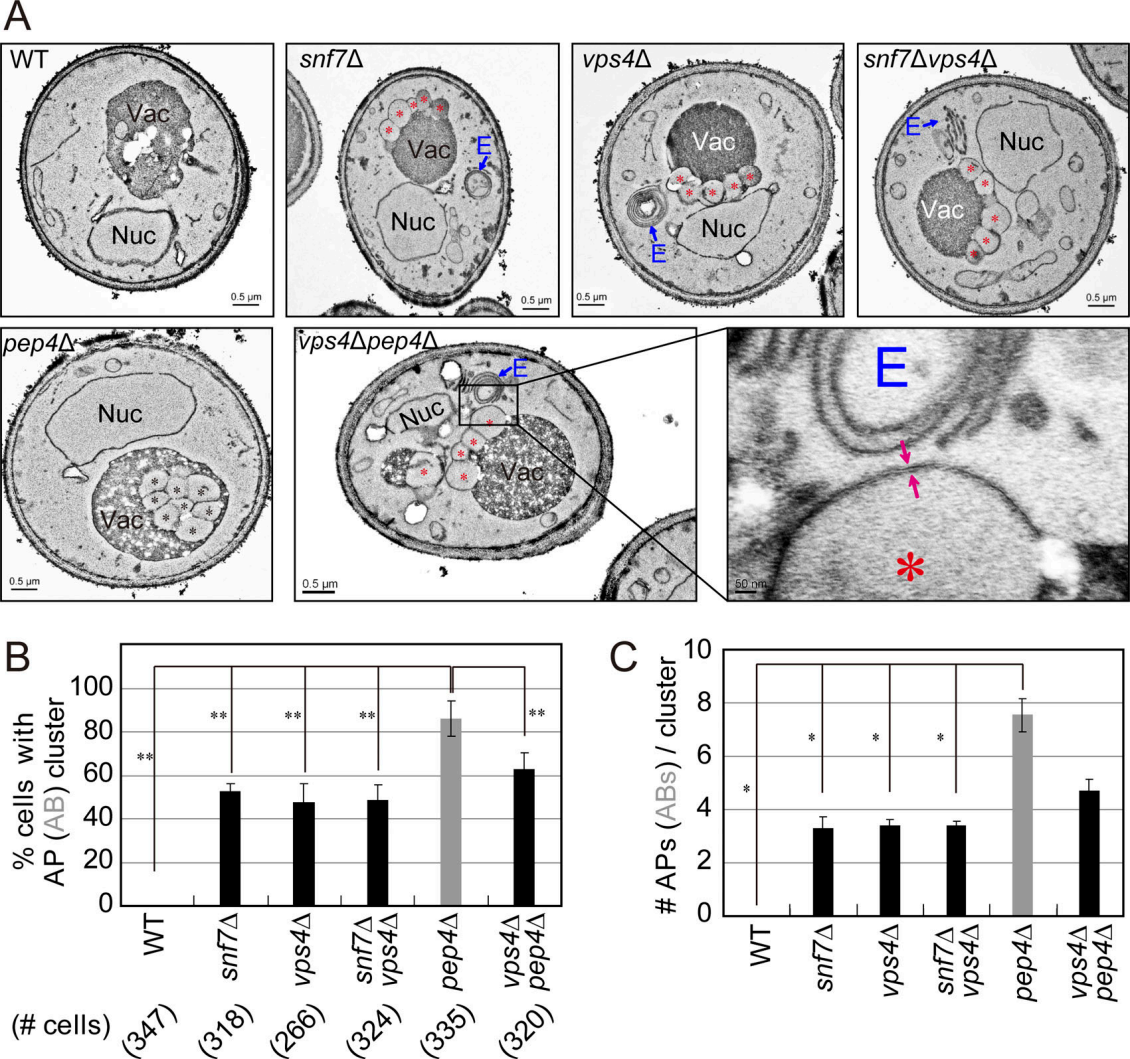
**Ultrastructure analysis of membrane structures that accumulate in ESCRT mutant cells. (A)** WT and mutant cells were starved for 2 h in SD-N (as in Fig. 2), fixed, and subjected to transmission EM. In addition to class E compartment, all single and double ESCRT mutant cells accumulate normal-sized APs (∼500 nm); red arrows point to the double membrane of an AP seen in the inset. Only *pep4Δ* single-mutant cells accumulate ABs inside their vacuole. E, class E compartment; Nuc, nucleus; Vac, vacuole; red asterisk, AP; black asterisk, AB. **(B and C)** Quantification of membrane structures that accumulate in mutant cells from A. **(B)** Percentage of cells with APs (black bars) or ABs (gray bar; bottom, number of cells used for quantification). **(C)** Number of APs (black bars) or ABs (gray bar) per cluster. Columns represent mean, error bars represent SD. Results in this figure represent three independent experiments. *P < 0.05; **P < 0.01.

Finally, EM analysis was used to confirm that APs accumulate in ESCRT mutant cells. Under starvation, ESCRT mutant cells accumulate multilamellar-cupped membrane structures (Fig. 3 A) typical of the class E compartment (Rieder et al., 1996). In addition, 50% of the *snf7Δ* and *vps4Δ* single-mutant cells accumulate membrane-surrounded structures in a cluster near their vacuole (∼3.5/cluster) with a size typical of APs, ∼500 nm. In high magnification, double membrane can be seen surrounding these structures, supporting the idea that they represent APs (Fig. 3 A, inset). Moreover, whereas ABs accumulate in >80% of the *pep4Δ* mutant cells (∼7/cell), they do not accumulate in *vps4Δ pep4Δ* double-mutant cells, which instead accumulate APs, like *vps4Δ* mutant cells (Fig. 3, A–C). Thus, the EM results agree with the live-cell fluorescence microscopy analysis (above). Furthermore, the AP accumulation phenotype in *snf7Δ vps4Δ* double mutants is similar to that of the *snf7Δ* and *vps4Δ* single mutants in terms of percentage of cells with APs and the average number of APs per cluster (Fig. 3). The finding that the partial phenotypes of *snf7Δ* and *vps4Δ* single mutants are not additive in the double mutant suggests that Snf7 and Vps4 function in the same pathway in autophagy.

Together, these results show that, like *vps21Δ* mutant cells, the ESCRT mutants *snf7Δ* and *vps4Δ* are defective in a late step of autophagy, following Atg1-mediated AP formation and before delivery of ABs to the vacuole where Pep4-dependent degradation occurs.

### ESCRT proteins mediate AP sealing

**Figure 4.**
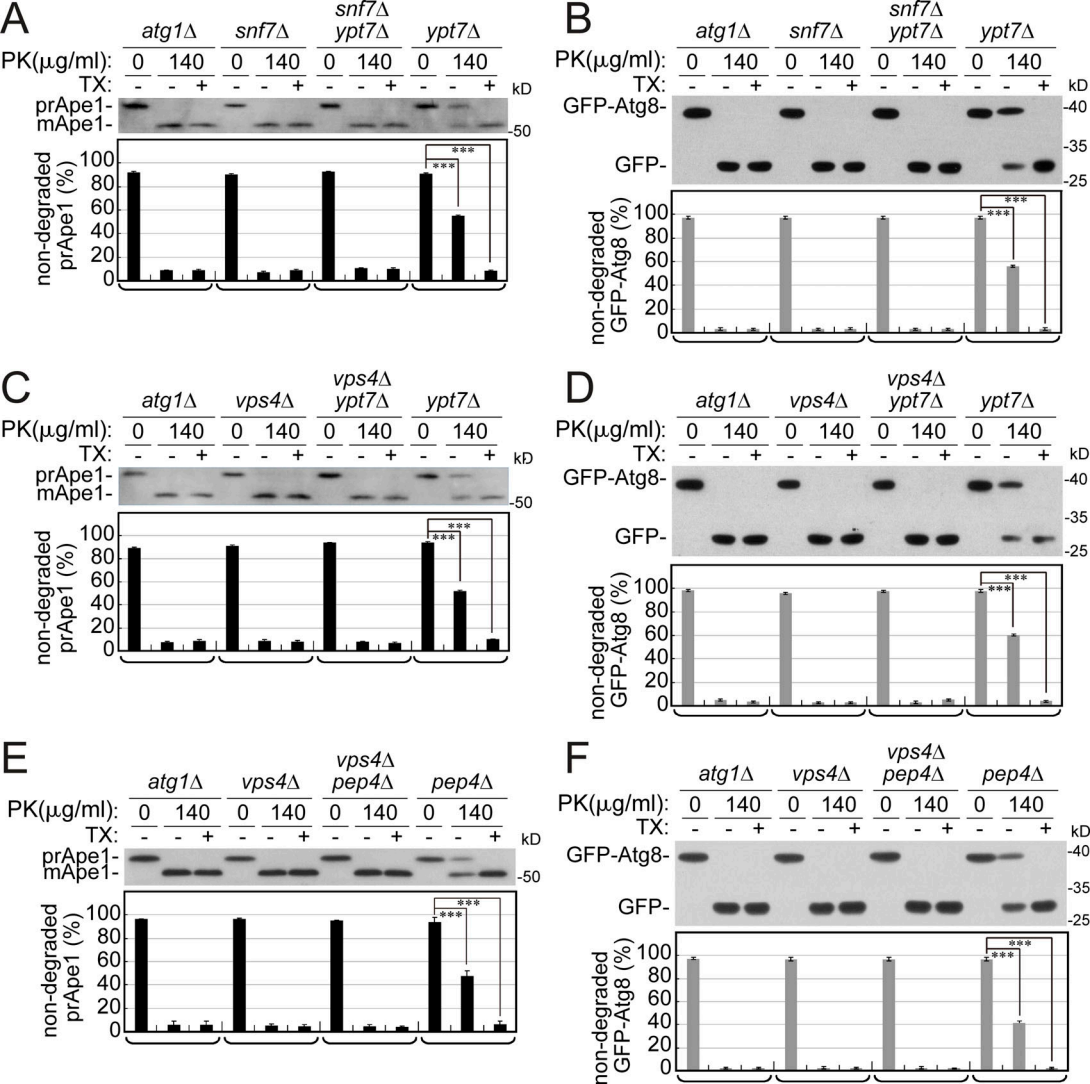
**Autophagy cargos that accumulate in single and double ESCRT mutant cells are completely sensitive to protease. (A and B)** *SNF7* is epistatic to *YPT7*. **(C and D)** *VPS4* is epistatic to *YPT7*. **(E and F)** *VPS4* is epistatic to *PEP4*. Lysates of WT and single- and double-mutant cells expressing GFP-Atg8 were subjected to the conventional protease (PK)-protection assay combined with immunoblot analysis (see Materials and methods). Levels of Ape1 (A, C, and E; black bars) and GFP-Atg8 (B, D, and F; gray bars) were determined before (0) and after addition of protease (PK), with or without addition of detergent (TX). Protease protection of cargos can be seen when PK is added to membranes without detergent (middle of each lane triplet). Nondegraded cargos: prApe1 and GFP-Atg8; degraded cargos: mApe1 and GFP. Bottom: Bar graph showing percentage of nondegraded cargos; columns represent mean and error bars represent SD. Results represent three independent experiments. ***P < 0.001.

Using a protease-protection assay combined with immunoblot analysis, we recently showed that APs that accumulate in *vps21Δ* mutant cells are open (Zhou et al., 2017). In this assay, a membrane fraction enriched for APs (P10) is purified from cell lysates and subjected to proteinase K (PK), and autophagy cargos are detected by immunoblot analysis. Here, this assay was used to analyze APs that accumulate in the ESCRT mutants *snf7Δ* and *vps4Δ*. In mutant cells defective in AP formation (e.g., *atg1Δ*), autophagy cargos are not protected from degradation by proteases. In contrast, in mutant cells that accumulate closed APs (e.g., *ypt7Δ*) or ABs inside the vacuole, like *pep4Δ*, 50–60% of the autophagy cargos are protected from degradation by proteases; this protection is abolished when detergent is added (Nair et al., 2011). Using two autophagy cargos, Ape1 and GFP-Atg8, we show that both cargos are completely sensitive for degradation in membrane fractions isolated from *snf7Δ* and *vps4Δ* mutant cells. Moreover, double-mutant analyses showed that *SNF7* and *VPS4* are epistatic to *YPT7*; namely, in *snf7Δ ypt7Δ* and *vps4Δ ypt7Δ* double-mutant cells, autophagy cargos are not protected (Fig. 4, A–D). Similarly, double-mutant analysis of *vps4Δ* and *pep4Δ* showed that *VPS4* is epistatic also to *PEP4* (Fig. 4, E and F). Thus, the conventional protease-protection assay in combination with a double-mutant analysis showed that APs that accumulate in *snf7Δ* and *vps4Δ* mutant cells (Fig. 2 and Fig. 3) are open and that the step mediated by Snf7 and Vps4 precedes the steps of Ypt7-mediated AP fusion with the vacuole, and Pep4-catalyzed AB degradation inside the vacuole.

**Figure 5.**
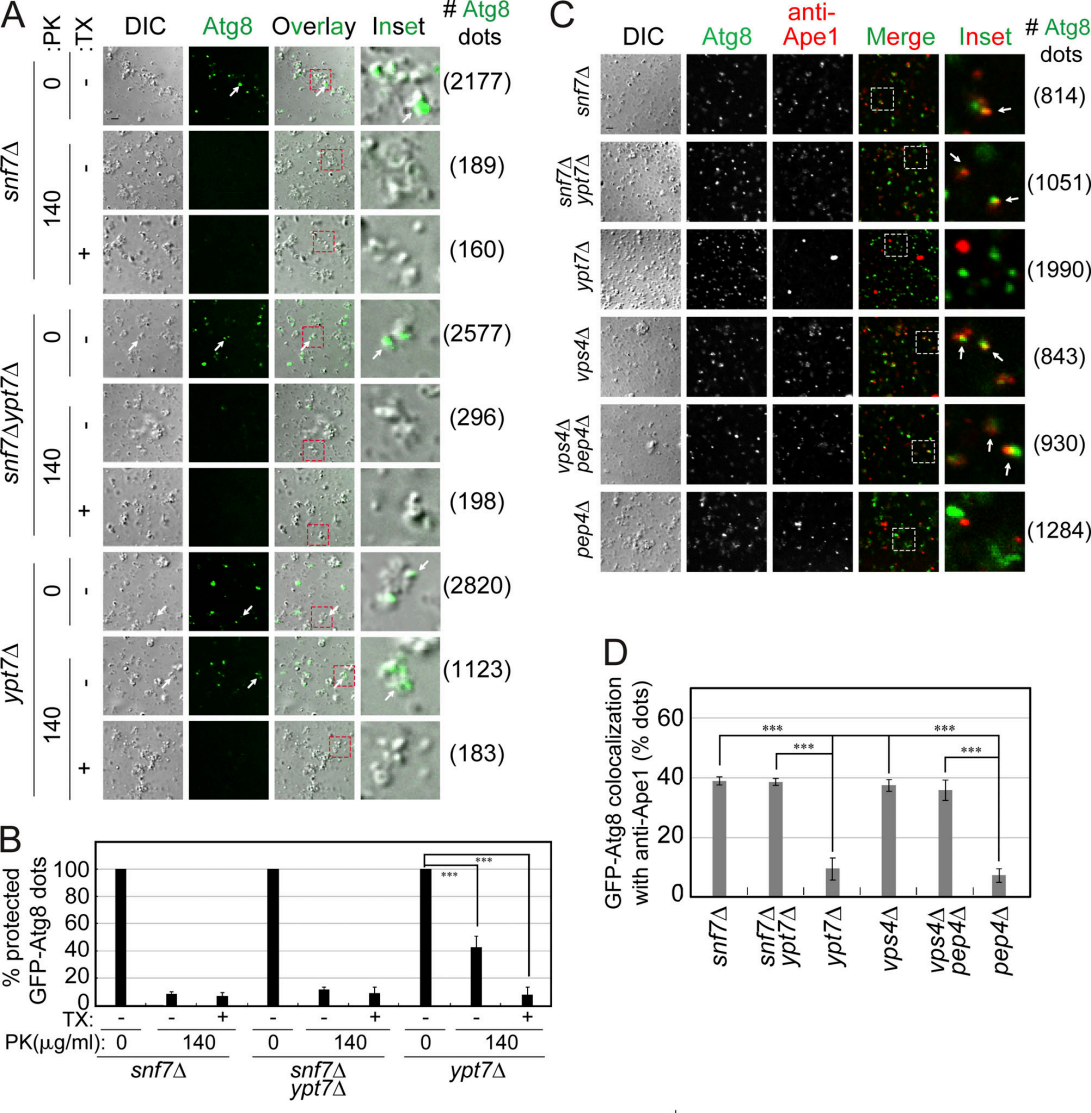
**Two new microscopy assays show that autophagy cargos accumulating in single and double ESCRT mutant cells are accessible to protease and anti-cargo antibodies. (A)** Modified protease-protection assay. Following the protease (PK) assay, fractions were washed to remove degraded peptides, and the presence of GFP-Atg8 in membrane fractions was determined by fluorescence microscopy (right, number of Atg8 dots used for quantification shown in B). Protease protection of GFP-Atg8 can be seen when PK is added to membranes without detergent. Scale bar, 2 µm. Arrows point to GFP-Atg8 positive dots/particles. **(B)** Bar graph shows percentage of GFP-Atg8 dots protected from the protease for the strains from A; >2000 DIC particles per condition were quantified (9 fields, 3 fields × 3 repeats). **(C)** Accessibility to antibodies against autophagic cargo assay. Membrane fractions from cells expressing GFP-Atg8 (for AP marking) were subjected to immunofluorescence microscopy using anti-Ape1 antibodies (right, number of Atg8 dots used for quantification in D). Particles (DIC) in which Ape1 and Atg8 colocalized show that the cargo was accessible to the antibodies (white arrows). Scale bar, 2 µm. **(D)** Bar graph showing GFP-Atg8 colocalization with anti-Ape1 (% of Atg8 dots) for the strains from C; >800 GFP-Atg8 dots per strain were quantified. In B and D, columns represent mean and error bars represent SD. Results in this figure represent three independent experiments. ***P < 0.001.

To support the idea that APs that accumulate in ESCRT mutant cells are open, we developed two new assays for distinguishing open and closed APs. The first is a modified protease-protection assay, but instead of immunoblot analysis the readout depends on visualization of the autophagy cargo GFP-Atg8 using fluorescence microscopy. Following the protease reactions, the presence of GFP-Atg8 that remains in the membrane samples after a wash was directly visualized by fluorescence microscopy. While in untreated samples GFP-Atg8 is seen in membrane structures (visualized by differential interference contrast [DIC]), PK can degrade the GFP-Atg8 if the APs are open but not if they are closed. In *ypt7Δ* mutant samples, ∼40% of the GFP-Atg8 dots remain after protease treatment. Fractions from all strains, including in *ypt7Δ*, have almost no GFP-Atg8 dots if detergent is added during the protease reaction, showing that the protection is provided by a membrane. Importantly, in membrane fractions from *snf7Δ* and *snf7Δ ypt7Δ* mutant cells, the same low background number of GFP-Atg8 dots remains after treatment with protease in the presence or absence of detergent (Fig. 5, A and B). Thus, results of the modified protease-protection assay agree with the traditional immunoblot-based assay that open APs accumulate in *snf7Δ* mutant cells and that the function of Snf7 precedes that of Ypt7.

In the second new assay, instead of determining accessibility of AP cargo to a protease, the assay depends on accessibility of this cargo to antibodies against it. The readout depends on microscopy for detection of anti–cargo antibodies present in APs. The cellular fraction used for the protease-protection assays (P10) was incubated with anti-Ape1 antibodies. The presence of the antibodies against the AP cargo Ape1 and their colocalization with the AP marker GFP-Atg8 was determined by immunofluorescence microscopy. In membrane fractions from *ypt7Δ* and *pep4Δ* mutant cells, in which the AP cargo is shielded from the antibodies by the AP or AB membranes, respectively, the level of Ape1 and GFP-Atg8 colocalization is very low (∼5%). In contrast, ∼40% of the GFP-Atg8 dots colocalize with Ape1 in fractions from *snf7Δ* and *vps4Δ* mutant cells. Thus, this assay shows that Ape1 that accumulates in APs from *snf7Δ* and *vps4Δ* mutant cells is accessible to antibodies against it. Moreover, in the double mutants *snf7Δ ypt7Δ* and *vps4Δ pep4Δ*, the colocalization is similar to that seen in the single ESCRT mutant cells (∼40%); namely, the ESCRT genes are epistatic to *YPT7* and *PEP4* (Fig. 5, C and D). These results agree with those of the protease-protection assays.

**Figure 6.**
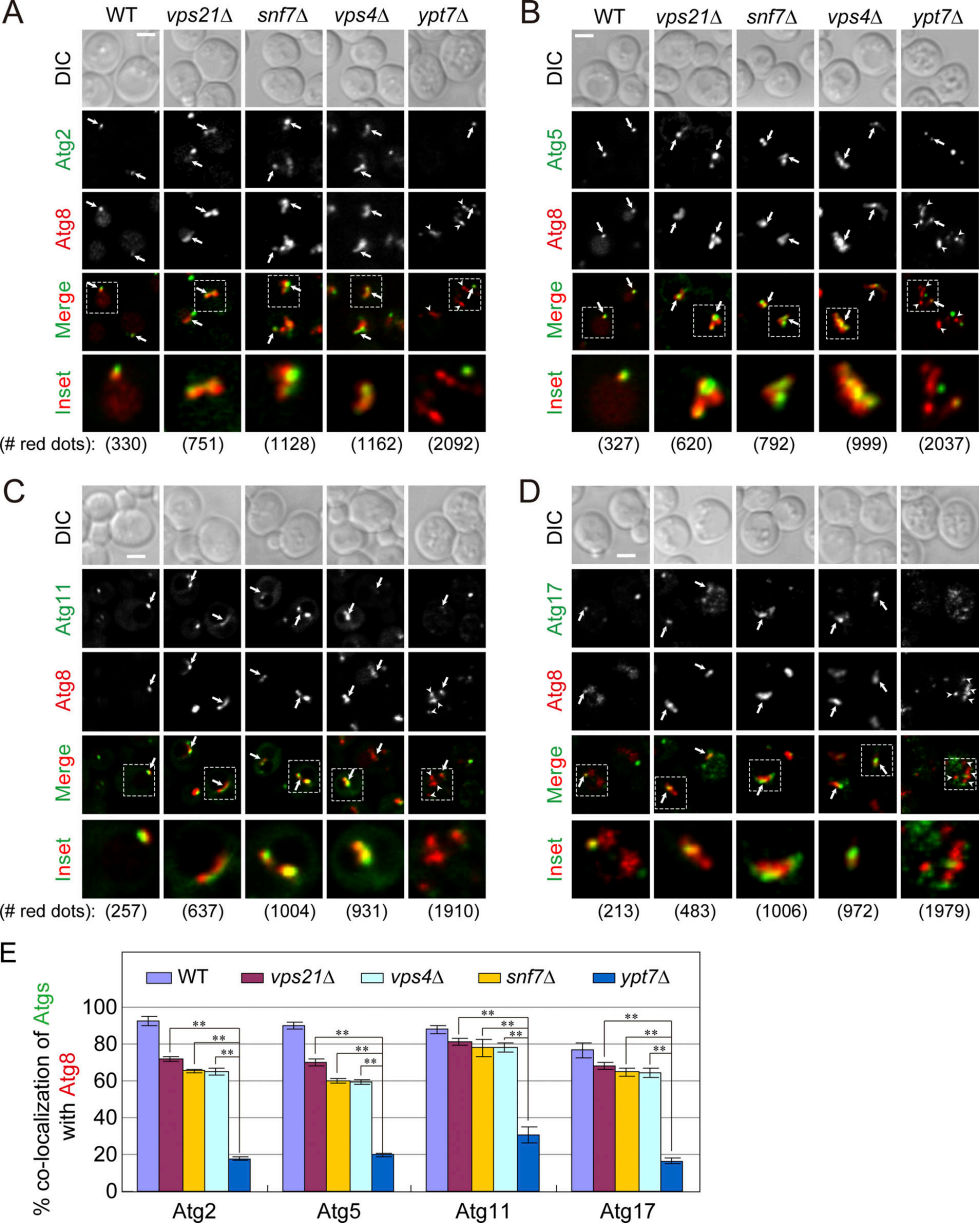
**Dissociation of Atg2, Atg5, Atg11, and Atg17 from accumulated APs is defective in *snf7*Δ and *vps4*Δ mutant cells. (A–D)** Endogenous Atg2 (A), Atg5 (B), Atg11 (C), and Atg17 (D) were tagged with GFP at their C terminus and Atg8 was tagged with mCherry at its N terminus in WT and mutant cells. Cells were grown in YPD medium and autophagy was induced by starvation (SD-N for starvation for 2 h; WT for 30 min). The colocalization of AtgX-GFP with mCherry-Atg8 was monitored using live-cell fluorescence microscopy (bottom, number of red puncta used for quantification). Arrows indicate AtgX-Atg8 colocalization; arrowheads indicate Atg8 puncta that do not colocalize with AtgX. Scale bar, 2 µm. **(E)** Quantification for AtgX-Atg8 colocalization (%) from A–D. In WT cells, a single dot per cell contains AtgX and Atg8 and represents a pre-autophagosomal structure or APs. More than 200 mCherry-Atg8 dots per strain were examined. Columns represent mean and error bars represent SD. Results represent three independent experiments. **P < 0.01.

Based on the three different assays used here, we conclude that APs that accumulate in *snf7Δ and vps4Δ* mutant cells are open. We have previously shown that immature open APs that accumulate in *vps21Δ* mutant cells are decorated with Atgs, while most Atgs are not present on closed APs that accumulate in *ypt7Δ* mutant cells (Zhou et al., 2017). If APs that accumulate in ESCRT mutant cells are open, we expected that they would be decorated with Atgs. The colocalization of Atg8, an AP marker that stays on the APs throughout their lives and after their fusion with the vacuole, with four Atgs (Atg2, Atg5, Atg11, and Atg17), which are removed from mature APs, was determined using live-cell fluorescence microscopy. While very low colocalization was observed between Atg8 and all four Atgs in *ypt7Δ* mutant cells (∼20%), 60–80% colocalization was seen in *snf7Δ* and *vps4Δ* mutant cells, similar to that seen in *vps21Δ* mutant cells (Fig. 6). The finding that APs that accumulate in ESCRT mutant cells are decorated with Atgs provides independent support to the idea that these APs are immature.

**Figure 7.**
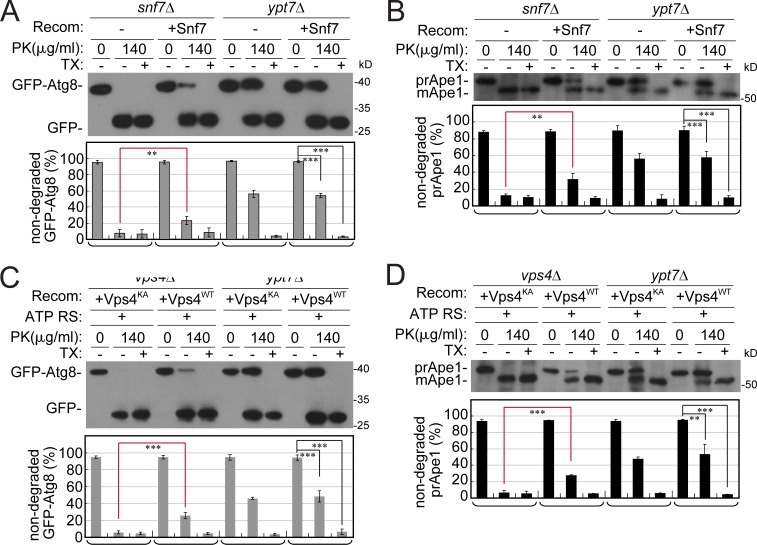
**Biochemical complementation of the AP closure defect of ESCRT mutants by purified recombinant proteins.** Membrane fractions from ESCRT mutants *snf7Δ* and *vps4Δ* were incubated with Snf7 or Vps4 purified from bacteria. Samples were then subjected to the protease-protection assay combined with immunoblot analysis (see Materials and methods). **(A and B)** Partial protection of the autophagy cargos GFP-Atg8 (A) and prApe1 (B) from protease (PK) in membrane fractions of *snf7Δ* mutant cells by Snf7 protein. **(C and D)** Partial protection of the autophagy cargos GFP-Atg8 (C) and prApe1 (D) from PK in *vps4Δ* mutant fractions by Vps4 WT, but not Vps4^K179A^ mutant protein. Vps4 or the ATPase-defective Vps4^K179A^ mutant protein was added in the presence of ATP regeneration system (ATP-RS). Columns represent mean, error bars represent SD. Results represent three independent experiments. **P < 0.01; ***P < 0.001. Recom, recombinant.

To support the idea that ESCRT machinery mediates AP sealing, we used a biochemical complementation assay. In this assay, purified recombinant proteins, Snf7 or Vps4, were added to membrane fractions (P10) from *snf7Δ* and *vps4Δ*, respectively, before the protease-protection assay was performed. For Vps4, we used the WT protein and a Vps4 mutant defective in its ATPase activity, Vps4^K179A^ (Babst et al., 1997), as a negative control; the ATPase activity of the WT, but not the mutant, protein was confirmed (Fig. S5, A and B). If the recombinant protein can complement the function missing in the mutant fraction and mediate AP sealing, we expected that some of the cargo that was accessible to protease in open APs would be protected when they are sealed. Ape1 and GFP-Atg8 were used as AP cargos. As shown above, AP cargos from *snf7Δ* mutant cells were completely sensitive to PK (same as the background seen when detergent was added). Importantly, a significant portion of the cargos (>20–30%) was protected from degradation when purified recombinant Snf7 was added to *snf7Δ* mutant fractions before testing the cargo for accessibility to PK (Fig. 7, A and B). Similarly, addition of WT Vps4 recombinant protein but not a Vps4 mutant defective in its ATPase activity (Vps4^K179A^) yielded ∼20% protected AP cargos in *vps4Δ* fractions, which are otherwise completely not protected (Fig. 7, C and D). Thus, recombinant ESCRT proteins can complement the AP sealing defects of their corresponding ESCRT mutants.

Together, results shown here indicate that *snf7Δ* and *vps4Δ* mutant cells accumulate open immature APs and Snf7 and Vps4 catalyze AP sealing.

### Rab5 GTPase regulates the localization of ESCRT subunits to APs

Our previously published results (Zhou et al., 2017), together with those presented above, show that depletion of the Rab5 GTPase Vps21 or the ESCRT subunits Snf7 and Vps4 results in accumulation of open APs. Whereas some autophagy phenotypes exhibited by these mutants are complete, their starvation-induced autophagy cargo-processing defects are partial. To confirm that Vps21 and the ESCRT machinery components function in the same starvation-induced autophagy pathway, we performed a double-mutant analysis of the partial phenotypes. If the two proteins function in the same pathway, we expect that the phenotype of the double mutant would not be more severe than those of the single mutants. In contrast, if they function in parallel pathways, the phenotypes would be additive. Processing of two autophagy cargos, GFP-Atg8 and Ape1, under starvation was determined in *vps21Δ* and *vps4Δ* single mutants and was compared with that of the *vps21Δ vps4Δ* double mutant. Like the single mutants, double-mutant cells exhibited partial autophagy defects for both cargos that were not more severe than those of the single mutants (Fig. S6 A). Similarly, the GFP-Atg8 cluster accumulation phenotype under starvation was not more severe in the *vps21Δ vps4Δ* double mutant when compared with the single mutants (Fig. S6 B). These results indicate that the Rab5 GTPase Vps21 and the ESCRT machinery function in the same autophagy pathway.

In endocytosis, Vps21 functions upstream of the ESCRT machinery (Russell et al., 2012). We wished to determine whether in autophagy Vps21 also functions upstream of ESCRT. One possibility is that Vps21 function is required for recruitment of ESCRT subunits to Aps, and we tested this for Snf7 and Vps4.

**Figure 8.**
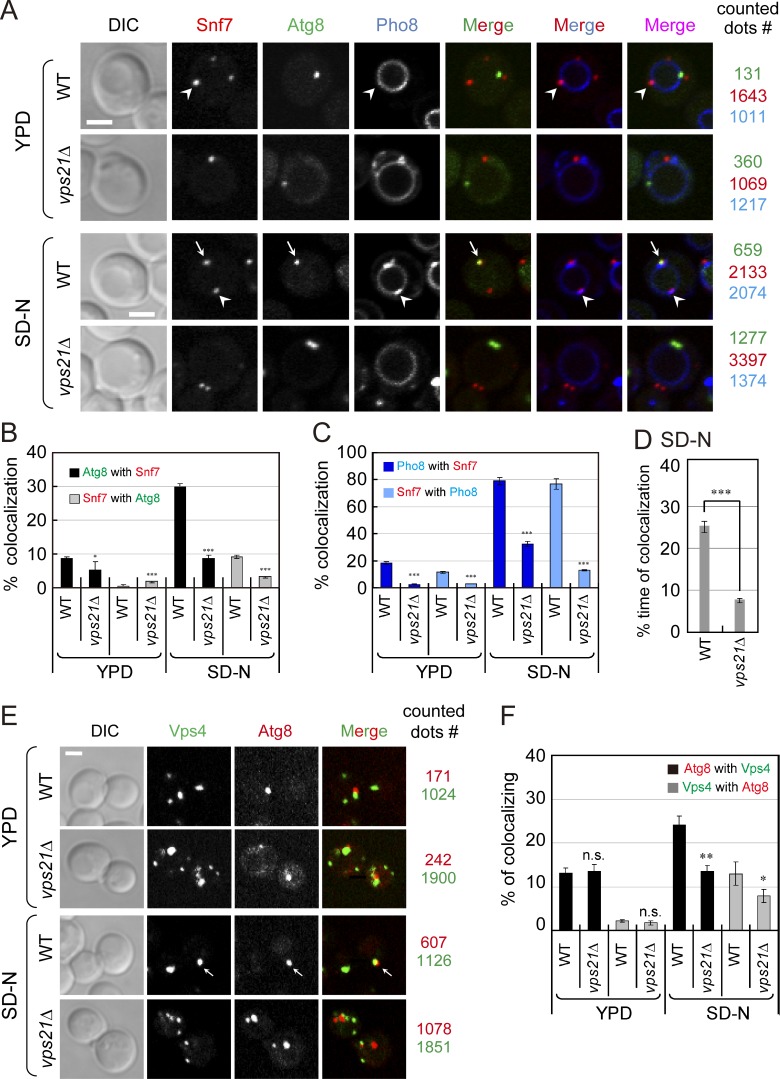
**Localization of the ESCRT subunits Snf7 and Vps4 to APs is regulated by the Rab5 GTPase Vps21. (A–C)** During starvation, Snf7 colocalizes with Atg8-marked APs and Pho8-marked class E compartment in a Vps21-dependent manner. **(A)** WT and *vps21Δ* mutant cells expressing GFP-Atg8, Snf7-mCherry, and 3×tagBFP-Pho8 were grown in YPD (top), shifted to SD-N (30 min, bottom), and visualized by live-cell fluorescence microscopy (right, number of dots counted for the quantification in B and C). Arrows point to Atg8-Snf7 colocalization; arrowheads indicate Snf7-Pho8 colocalization. Scale bar, 2 µm. **(B)** During starvation, Snf7 localization to APs is lower in *vps21Δ* mutant cells when compared with WT cells. Bar graph showing percentage of colocalization of Atg8 with Snf7 (black bars) and Snf7 with Atg8 (gray bars). **(C)** During starvation, the majority of Snf7 colocalize with Pho8. This colocalization is also lower in *vps21Δ* mutant cells when compared with WT cells. Bar graph showing percentage of colocalization of Pho8 with Snf7 (dark blue bars) and Snf7 with Pho8 (light blue bars). More than 100 GFP-Atg8, Snf7-mCherry, or 3×tagBFP-Pho8 dots per strain were examined in B and C. **(D)** During starvation, Snf7 localizes to APs in *vps21Δ* mutant cells and for about one third of the time it does so in WT cells. Quantification of time-lapse video microscopy (6-s intervals during 25 min) showing time points (%) in which the two proteins colocalized (Videos 3 and 4). 30 cells per strain were quantified. **(E and F)** During starvation, Vps4 colocalizes with the Atg8-marked APs in a Vps21-dependent manner. **(E)** WT and *vps21Δ* mutant cells expressing endogenously tagged Vps4-NG and mCherry-Atg8 were grown in YPD (top) or SD-N (bottom) and visualized as described for A (right, number of dots counted for the quantification in F). Arrows point to Atg8-Vps4 colocalization. Scale bar, 2 µm. **(F)** During starvation, Vps4 localization to APs is lower in *vps21Δ* mutant cells when compared with WT cells. Bar graph showing percentage of colocalization of Atg8 with Vps4 (black bars) and Vps4 with Atg8 (gray bars). Columns in B–D and F represent mean; error bars represent SD. Results represent three independent experiments. n.s., not significant; *P < 0.05; **P < 0.01; ***P < 0.001.

Snf7 was tagged at its C terminus with mCherry (and was expressed under its own promoter and terminator from the *TRP1* locus). Snf7-mCherry is functional because *snf7Δ* mutant cells expressing it as the only Snf7 copy do not accumulate class E compartment during normal growth, and under starvation these cells deliver Atg8 to the vacuole like WT cells, and do not accumulate APs like the *snf7Δ* mutant cells (Fig. S5, C and D). The localization of Snf7-mCherry to APs marked by GFP-Atg8 was compared in WT and *vps21Δ* mutant cells. During normal growth of WT and *vps21Δ* mutant cells, <10% of Atg8 puncta, which represent preautophagosomal structure or APs, contain Snf7. Under starvation, even though most of Snf7 localizes to class E compartment (marked with Pho8, >70%), its localization to APs is increased to 30% in WT cells but not in *vps21Δ* mutant cells (Fig. 8, A–C). The localization of Snf7 to APs in WT cells is expected to be transient and the observed ∼30% colocalization of Atg8 with Snf7 is thus sufficient for Snf7 function.

To assess the dynamic localization of Snf7 to APs we used time-lapse microscopy. The colocalization of Snf7 with the APs in WT cells is dynamic: in ∼25% of the time points taken for a single cell colocalization was observed (Video 3). The observed significant reduction in the colocalization of Snf7 and Atg8 in *vps21Δ* mutant cells is due to less time in which the two proteins colocalize (Video 4 and Fig. 8 D). These results show that the increase in Snf7 localization to APs during starvation is controlled by the Rab5 GTPase Vps21. In fact, Vps21 controls the recruitment of the ESCRT III subunit Snf7 to both endosomes and APs where ESCRT mediates ILV formation and AP sealing, respectively (Fig. 8, A–C).

Similar results were observed for Vps4 localization to APs. Endogenous Vps4 was tagged with NG at its C terminus. Vps4-NG is functional because when expressed as the only copy of Vps4, the cells do not accumulate class E compartment during normal growth like *vps4Δ*, and under starvation Atg8 is delivered to the vacuole as in WT cells, and AP clusters do not accumulate as in *vps4Δ* mutant cells (Fig. S5, E and F). The localization of Vps4-NG to APs marked by mCherry-Atg8 is similar to that of Snf7: up from ∼10% during normal growth to ∼25% during starvation in WT cells, but not in *vps21Δ* mutant cells (Fig. 8, E and F). Thus, Vps21 controls the localization of the two ESCRT subunits Snf7 and Vps4 to APs during starvation.

### Rab5-dependent Atg17–Snf7 interaction is important for autophagy

**Figure 9.**
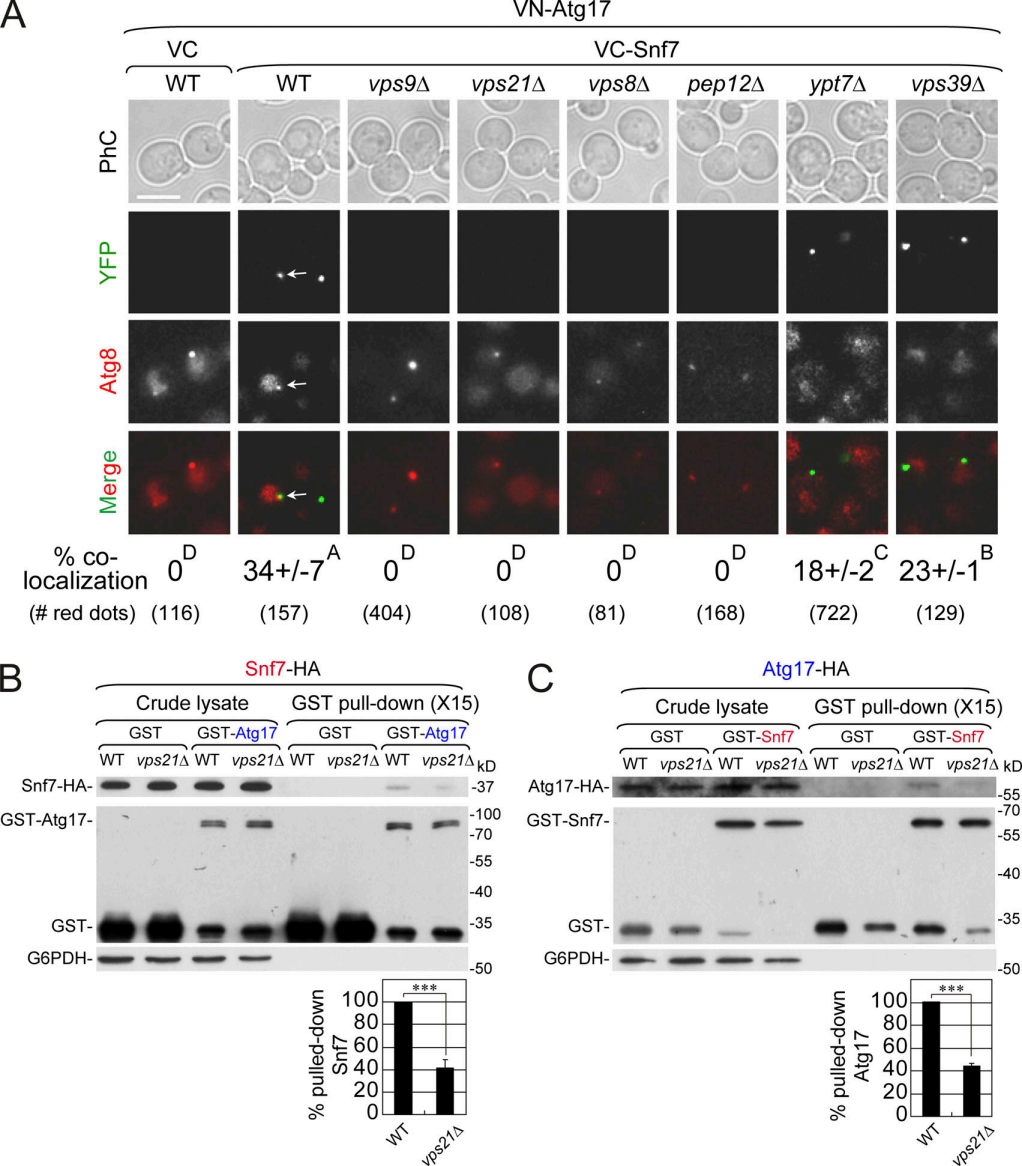
**Two different assays reveal a Rab5-dependent Atg17–Snf7 interaction. (A)** Vps21-dependent Atg17–Snf7 BiFC interaction occurs on APs. WT and mutant cells expressing mCherry-Atg8 from the chromosome and VN-Atg17 from a plasmid were cotransformed with VC plasmids: VC empty (left panel) or VC-Snf7 (rest of panels). Cells were shifted to SD-N (30 min) before YFP fluorescence was observed using live-cell fluorescence microscopy (bottom, percentage of cells with YFP dots colocalizing with Atg8, and number of red dots used for the analysis). Scale bar, 5 µm. More than 80 cells per strain were examined; data are presented as the mean ± SD of each variable from three independent experiments. The same capital letter at the top right corner of each mean ± SD indicates no statistically significant difference, while different capital letters indicate significant difference (P < 0.05). **(B and C)** Vps21-dependent GST-pulldown of Atg17 and Snf7 with each other. WT and *vps21Δ* mutant cells expressing GST as a negative control, GST-Atg17 (from a plasmid), and endogenously tagged Snf7-HA (B) or GST-Snf7(from a plasmid) and endogenously tagged Atg17-HA (C) were grown, shifted to SD-N (30 min), lysed (crude lysate, left), and subjected to a GST-pulldown analysis (right) followed by Western blot analysis (see Materials and methods). Bar graphs (bottom) show percentage of HA-tagged Snf7 (B) or Atg17 (C) that were pulled down by GST-Atg17 or GST-Snf7, respectively, from WT (100%) and *vps21Δ* mutant cell lysates. Columns represent mean, error bars represent SD. Results represent three independent experiments. ***P < 0.001.

To understand the mechanism by which Rab5 regulates ESCRT recruitment we explored possible interactions of Snf7 with Atgs and found that it interacts with some subunits of the Atg1 complex that act in early autophagy (Davies et al., 2015). In a bi-fluorescence interaction complementation (BiFC) assay, Snf7 exhibits interaction with Atg11 and Atg17 (in >25% of cells) and a weak interaction with Atg29 (∼8% of cells), but not with Atg1, Atg13, or Atg31 (Fig. S7 A). We focused on the Atg17–Snf7 interaction because, unlike Atg11, which is specific for selective autophagy, Atg17 is required for stress-induced autophagy (Weidberg et al., 2011). YFP dots representing the BiFC interaction were seen only when cells were transformed with plasmids expressing VN-Atg17 and VC-Snf7, but not empty VC; and in WT cells, 35% of the Atg8-marked APs also contain YFP (Fig. 9 A). The domain required and sufficient for the BiFC interaction with Atg17 is the membrane-binding domain of Snf7 (Tang et al., 2015; Fig. S7, B and C). Importantly, the Atg17–Snf7 BiFC interaction is dependent on members of the Rab5 GTPase module, including the Vps9 GEF, Vps21 GTPase, and Vps8 and Pep12 effectors, but not on Ypt7 GTPase and its effector Vps39 (Fig. 9 A).

GST pulldown from yeast cell lysates was used as an independent assay to confirm the Atg17–Snf7 interaction. Pulldowns were done with lysates from cells expressing one protein tagged with GST from a plasmid and the other tagged with HA in its endogenous locus. When the analysis was done in WT cells, the HA-tagged protein was pulled down with the GST-tagged protein in a reciprocal way. Pulldown of both Snf7-HA with GST-Atg17 and Atg17-HA with GST-Snf7 was significantly lower in *vps21Δ* mutant cells (∼40% of the interaction observed in WT cells; Fig. 9, B and C). Thus, two independent assays, BiFC and reciprocal coprecipitations, show that Snf7 interacts with Atg17 in a Rab5-dependent manner.

**Figure 10.**
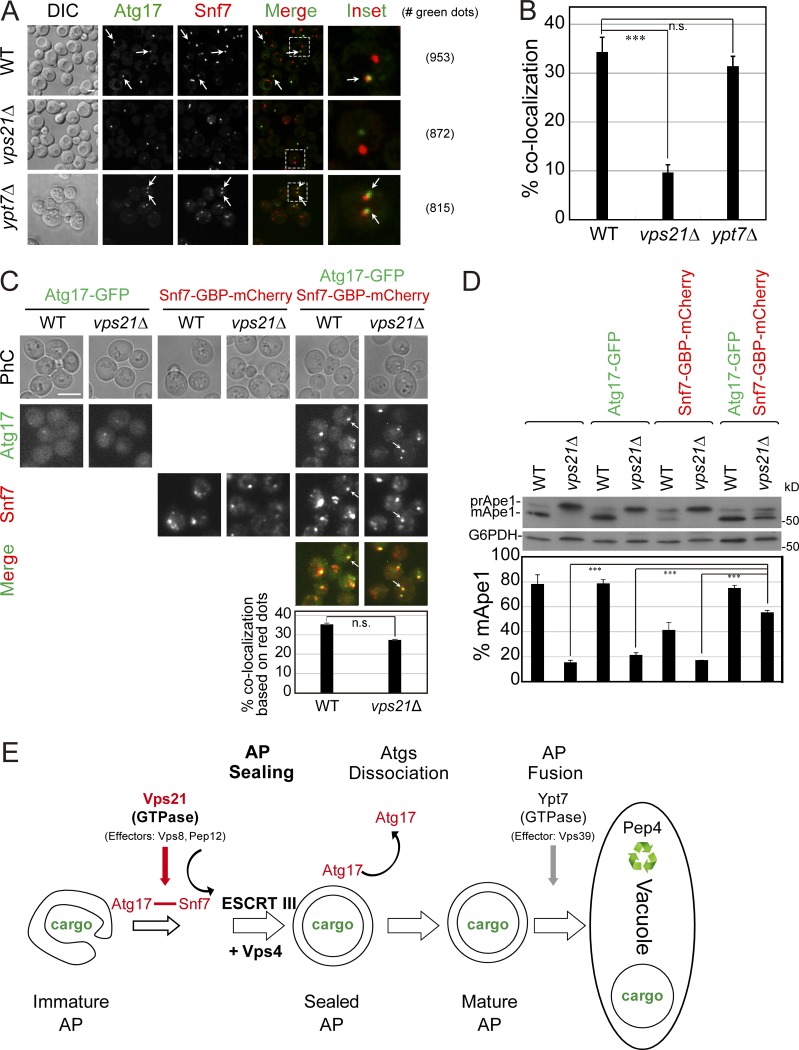
**Atg17 and Snf7 colocalize in a Vps21-dependent manner and forcing their interaction bypasses the requirement for Vps21 GTPase in autophagy. (A and B)** Atg17 and Snf7 colocalize in a Vps21-dependent manner. WT and mutant cells expressing Atg17-GFP and Snf7-mCherry were grown to mid-log phase, shifted to SD-N for 30 min, and analyzed by live-cell fluorescence microscopy (A; right, number of green dots used for quantification in B). **(C)** Coexpression of Atg17-GFP and Snf7-GBP-mCherry results in their colocalization even in *vps21Δ* mutant cells. WT and *vps21Δ* mutant cells expressing from their endogenous loci Atg17-GFP (left), Snf7-GBP-mCherry (middle), or both (right) were grown, starved, and visualized as described for A. Bar graph showing percentage of red dots colocalizing with green dots in WT and *vps21Δ* mutant cells (bottom). **(D)** Forcing the Atg17–Snf7 interaction using GFP and GBP suppresses the autophagy defect of *vps21Δ* mutant cells. Processing of Ape1 in lysates from cells used in C was determined by immunoblot analysis; bar graph shows the percentage of processed mApe1 in the different cells. **(A and C)** Scale bar, 5 µm; arrows point to colocalizing puncta. **(B and D)** Bars represent mean ± SD of each variable from three independent experiments. Results represent three independent experiments. **(E)** Diagram of Rab5 GTPase-regulated and ESCRT III–catalyzed AP closure. We propose that a Rab5 GTPase module, which includes a GEF and downstream effectors (e.g., Vps8 and Pep12), controls the recruitment of ESCRT to unsealed APs through an interaction of the ESCRT subunit Snf7 with Atg17 on open APs. ESCRT machinery then catalyzes AP closure followed by Atgs dissociation (including Atg17) to yield mature APs, which, in turn, fuse with the lysosome in a Ypt7-regulated manner. Following fusion, ABs are deposited to the lysosomal lumen and autophagy cargos can be degraded by proteases (e.g., Pep4). n.s., not significant; ***P < 0.001.

Because the interaction occurs through the membrane-binding domain of Snf7, we tested whether Atg17 and Snf7 colocalize in a Rab5-dependent manner. The colocalization of Atg17-GFP and Snf7-mCherry expressed from their endogenous locus was determined during nitrogen starvation using live-cell microscopy. While in WT and *ypt7Δ* mutant cells the two proteins colocalize in ∼30% of the cells, the colocalization in *vps21Δ* mutant cells is reduced by threefold (Fig. 10, A and B).

To determine whether the Atg17–Snf7 interaction is important for autophagy, we tested the effect of an artificially forced interaction on the autophagy defect of *vps21Δ* mutant cells. The interaction was forced by adding a GFP-binding protein (GBP; Rothbauer et al., 2008) to the mCherry-tagged Snf7 and expressing it in the presence of Atg17-GFP. When Snf7-GBP-mCherry and Atg17-GFP were coexpressed from their endogenous loci, they colocalized under starvation in WT cells to the same extent as Atg17-GFP and Snf7-mCherry (>30%; compare B and C of Fig. 10). This level of colocalization is also comparable to that of Snf7 and Atg8-marked APs (Fig. 8, A and B). Importantly, while Atg17-GFP and Snf7-mCherry do not localize efficiently in *vps21Δ* mutant cells (Fig. 10, A and B), addition of the GBP tag to Snf7-mCherry forces their interaction and colocalization similarly in WT and *vps21Δ* mutant cells (Fig. 10 C). This shows that Snf7 can be forced to interact with Atg17 in a Vps21-independent manner using a GFP nano-trap.

The question was whether the forced interaction can bypass the autophagy defect of *vps21Δ* mutant cells. Upon nitrogen starvation, processing of prApe1 to mApe1 is about fourfold lower in *vps21Δ* mutant cells (∼20%) than in WT cells (∼80%). Expression of Snf7-GBP-mCherry or Atg17-GFP individually had no effect on Ape1 processing in *vps21Δ* mutant cells, while Snf7-GBP-mCherry itself had a mild negative effect on this processing in WT cells (possibly due to competition with the endogenous Snf7). Remarkably, while coexpression of Snf7-GBP-mCherry and Atg17-GFP together has no effect on Ape1 processing in WT cells, it suppresses the *vps21Δ* mutant cell phenotype (from ∼20% to >50% processing; Fig. 10 D). Thus, forcing the Atg17–Snf7 interaction artificially bypasses the requirement of the Rab5 GTPase Vps21 in autophagy.

## Discussion

Here, we show that the ESCRT machinery plays a role in a late step of the autophagy pathway in yeast, the ERCRT III subunit Snf7 and the Vps4 ATPase catalyze AP closure, and the Rab5 GTPase Vps21 controls the recruitment of Snf7 and Vps4 to APs. Moreover, we uncover a mechanism in which Rab5 regulates an interaction between Atg17 and Snf7 to recruit Snf7 to Atg17-decorated APs. These results settle the longstanding debate about a role for ESCRT in autophagy in yeast and answer the question of how AP closure is catalyzed. Based on our results, we delineate a pathway in which the Rab5 GTPase Vps21 recruits the ESCRT III subunit Snf7 to open APs decorated by Atg17, where the ESCRT machinery catalyzes AP closure. This step precedes Atg dissociation, Ypt7 GTPase-regulated AP fusion with the lysosome/vacuole, and Pep4 protease-dependent degradation of autophagic cargos (Fig. 10 E).

### ESCRT mutant cells exhibit late autophagy defects

We show that cells deleted for the ESCRT III subunit Snf7 and the Vps4 ATPase exhibit complete cargo-processing defects in the constitutive CVT pathway and severe phenotypes in starvation-induced cell death and alkaline phosphatase activity, similar to those of *atg1Δ* mutant cells. ESCRT mutant cells exhibit partial defects in processing of autophagy cargo during starvation, which cannot be explained by malfunctioning lysosomes as ESCRT mutant cells accumulate APs; namely, they exhibit a block before the autophagy cargo is delivered to lysosomes. Moreover, even though ESCRT mutant cells can process some, but not all, autophagy cargos, they still lose their viability during starvation like *atg1Δ* mutant cells, which exhibit a complete block in processing of autophagy cargo.

ESCRT deletion mutants being defective in a late autophagy step is supported by live-cell fluorescence microscopy combined with EM showing the accumulation of clusters of normal-size APs. Moreover, double-mutant analyses indicate that Snf7 and Vps4 function together downstream of Atg1-dependent AP formation, and before Ypt7-mediated AP fusion with the vacuole and the subsequent Pep4-dependent cargo degradation (Fig. 10 E).

We propose that previous studies of ESCRT in yeast autophagy have been inconclusive because, as we report, the severity of ESCRT mutant autophagy phenotypes depends on assay type, growth conditions, and the ESCRT subunit tested. For example, we showed that ESCRT mutants exhibit severe autophagy phenotypes in some, but not all, assays; that the severity of the AP-accumulation phenotypes depends on the length of the time they are grown in log phase before starvation (Zhou et al., 2017); and that not all ESCRT subunits play a role in autophagy (Fig. S1 and Fig. S2). Furthermore, partial defects in cargo processing that ESCRT mutants exhibit during autophagy could be due to open APs reaching the vacuole in an ESCRT-independent way (e.g., micro-autophagy).

### ESCRT subunits catalyze AP closure

Evidence presented here establishes that ESCRT machinery catalyzes AP closure. First, three different biochemical assays show that APs that accumulate in two ESCRT deletion mutants, *snf7Δ* and *vps4Δ*, are unclosed. Previously, the only method to detect unclosed APs in yeast was a protease-protection assay of autophagy cargos that uses immunoblot as a readout (Nair et al., 2011); therefore, we developed two new assays. The first uses protease protection with microscopy as a readout, while the second relies on antibody accessibility of autophagy cargo, analyzing colocalization of these antibodies with an AP marker as a readout by immunofluorescence microscopy. These assays should be useful for future experiments not only in yeast.

Second, the AP-closure defects exhibited by ESCRT mutant cells can be complemented in vivo by expressing the cognate protein from a plasmid and in vitro. Importantly, recombinant ESCRT proteins, Snf7 and Vps4, can complement the AP-closure defects of the ESCRT mutants *snf7Δ* and *vps4Δ*, respectively. In these assays, incubation of mutant membrane fractions with Snf7 and the Vps4 ATPase (but not a Vps4 mutant protein defective in the ATPase activity) before the protease-protection assay results in protection of autophagy cargos, which are otherwise completely unprotected.

Third, in vivo analysis supports the idea that APs that accumulate in ESCRT mutants are immature. One hallmark of immature APs is that they are decorated with Atgs, and these Atgs dissociate from closed mature APs that accumulate in *ypt7Δ* mutant cells (Reggiori and Ungermann, 2017). Live-cell fluorescence microscopy showed that APs that accumulate in *snf7Δ* and *vps4Δ* mutant cells, like those that accumulate in *vps21Δ* mutant cells (Zhou et al., 2017), are decorated with Atgs. Thus, ESCRT functions in autophagy before AP closure and Atg removal.

While we can distinguish between open and closed APs using biochemical assays, they look similar using EM (Fig. 3). This can be explained by the small opening needed for entry of a protein (protease or antibody), which is below the limit of resolution needed in the microscopy we have used here. In addition, there is no reason to expect that open and closed APs differ in their density. Thus, visualizing AP openings would require more sophisticated microscopy analysis.

### ESCRT role in late autophagy: Direct or indirect?

Establishing a direct role for ESCRT in autophagy has been challenging because of its importance in lysosomal functioning, the final destination of the endocytic and autophagy pathways (Müller et al., 2015). Alone, cargo-processing defects could be explained by indirect effects of malfunctioning vacuoles. Importantly, using single- and double-deletion mutant, genetic complementation, and localization analyses, we establish that ESCRT plays a role in a late step in autophagy, before autophagy cargo reaches the vacuole. The fact that the ESCRT-mediated step precedes the delivery of autophagic cargo to the lysosome argues against an indirect effect of ESCRT depletion due to defective vacuolar function.

Second, while both ESCRT and Ypt7 are required for endocytosis and lysosomal function, their depletion results in completely different autophagy phenotypes: accumulation of open APs decorated with Atgs and closed APs without Atgs, respectively. If their effect on autophagy was indirect, reflecting a backlog in the autophagy pathway due to impaired lysosomal protease function, they would be expected to have similar autophagy phenotypes. Thus, the different autophagy phenotypes exhibited by ESCRT and Ypt7 depletion support the idea that they both play a direct role in autophagy.

Third, the localization of functional Snf7 and Vps4 to APs is increased by two- to threefold under stress, as compared with normal growth. Thus, ESCRT mutant cells are defective in autophagy and at least two ESCRT subunits localize to APs during this process. These data provide additional support for a direct role of ESCRT in autophagy.

Last, we show that addition of ESCRT recombinant proteins, Snf7 and Vps4, to the corresponding mutant cellular fractions can complement their AP closure defect. This biochemical complementation strongly supports a direct role for ESCRT in autophagy.

Together, our cumulative data support a direct role for ESCRT in autophagy in addition to its role in endocytosis. In both pathways, ESCRT catalyzes a topologically similar reaction: membrane scission. The products of this scission event are MVBs in endocytosis and closed APs in autophagy.

### Rab5 GTPase regulates ESCRT recruitment to APs via Atg17–Snf7 interaction

Mutations in the Rab5 GTPase Vps21 (Chen et al., 2014; Zhou et al., 2017) and ESCRT subunits (this study) result in partial autophagy cargo-processing defects and a similar block in AP closure. Therefore, it was important to establish that Vps21 and ESCRT function in the same autophagy pathway. This was done using double-mutant analysis and showing that combining the two mutations, *vps21Δ* and *vps4Δ*, does not result in autophagy phenotypes that are more severe than those of the single mutations.

Because Rab GTPases are organizers of specific membrane domains (Zerial and McBride, 2001), we hypothesized that Rab5 regulates the recruitment of ESCRT to APs. Indeed, we show that the localization of the ESCRT subunits Snf7 and Vps4 to APs is regulated by Vps21 and so is the interaction of Snf7 with Atg17; the latter plays a role in AP formation and resides on APs until after their closure. Importantly, forcing the interaction of Atg17–Snf7 artificially can bypass the requirement of Vps21 for autophagy. Thus, our results support a mechanism in which Rab5 GTPase controls an Atg17–Snf7 interaction for recruitment of Snf7 and ESCRT to unclosed APs, where ESCRT catalyzes AP closure (Fig. 10 E). In early autophagy, Atg17 functions in the context of an Atg1 complex (Davies et al., 2015). Using the BiFC assay, we found that, in addition to Atg17, Snf7 interacts with two other subunits of this complex. Therefore, it would be important to determine whether Snf7 and Atg17 interact in the context of the Atg1 complex, or in the context of a separate complex that acts in late steps of autophagy.

### Future directions

Like Atgs, ESCRT is a conserved cellular machinery (Henne et al., 2013), and findings here would likely pertain to human cells. Indeed, during the final preparation of this manuscript, using knockdown of CHMP2A, the homologue of the ESCRT III subunit Vps2, and a dominant-negative Vps4 mutant, ESCRT was suggested to function in AP closure in human cells (Takahashi et al., 2018). Importantly, the role of Rab5 in ESCRT recruitment to open APs and the specific mechanism we delineated in yeast will have to be explored in human cells. Both Atgs and ESCRT were implicated in human diseases (Alfred and Vaccari, 2016; Dikic and Elazar, 2018) and connecting these two sets of machineries to the same process should have implications on understanding these diseases.

ESCRT machinery has been implicated in both micro- and macro-autophagy (Lefebvre et al., 2018). Usually, these two processes are viewed as parallel, with the first mostly independent of Atgs and APs (Galluzzi et al., 2017). Our findings that Rab5 and ESCRT mutants exhibit a partial block in autophagy cargo processing while accumulating open APs raises the possibility that in these mutant cells some open APs reach the lysosomal lumen in a Rab5- and ESCRT-independent manner. It is possible that micro-autophagy is involved in direct engulfment of open APs by endosomal or lysosomal membrane, providing an example in which micro- and macro-autophagy pathways intersect. Alternatively, there might be another way by which open APs can fuse with the lysosome.

Our results show that representatives from each of the four ESCRT complexes (0–III) play a role in a late step of autophagy. This suggests that not just membrane scission catalyzed by ESCRT III and the Vps4 ATPase is shared between AP closure and ILV formation in late endosomes but also processes like clustering of ubiquitinated proteins on membrane domains mediated by ESCRT 0–II. It would be important to inquire whether these ESCRT-dependent processes also occur during autophagy and determine whether other ESCRT subunits are recruited to APs by Rab5 GTPase or by its downstream effectors. Other future questions should address mechanisms by which other ESCRT subunits are recruited to APs. In addition, it is interesting why not all ESCRT subunits play a role in autophagy. The subunits that do not play a role in autophagy might provide clues in distinguishing between membrane scission of endocytic ILVs and APs.

## Materials and methods

### Strains, plasmids, and reagents

Yeast strains, plasmids, and oligos used in this study are listed in Table S1. Yeast and *Escherichia coli* transformations were performed as previously described (Liang et al., 2007). Reagents used in this study are as previously described (Zhou et al., 2017).

Strain and plasmid construction was done as follows: Gene deletions were obtained by replacing the complete ORF of targeted genes by the indicated cassettes using PCR-based homologous recombination. Strains expressing GFP-tagged endogenous Atgs at the C terminus were constructed by PCR-based homologous recombination as previously described (Zhou et al., 2017). pRS306-GFP-Atg8 or pRS304-mCherry-Atg8 was integrated in SEY6210 as previously described (Zhou et al., 2017). pRS305-*PGK1p-*3×tagBFP-Pho8 was linearized by BglII and integrated in GFP-Atg8 tagged SEY6210. NG*-*Cps1 was cloned into pRS416 vector and expressed under the *CPY* promoter to get pRS416-CPYp*-*NG*-*Cps1. ClhN-*SNF7*-mCherry-*TRP1* plasmid was linearized with Mfe1 at the promoter region of Snf7 and integrated into WT and *snf7Δ* mutant cells (in which GFP-Atg8 is expressed or not expressed) by selection on SD-Trp. ORF of *SNF7* was cloned under Adh81 promoter of pUC119-*Padh81*-GBP-mCherry(C)-hphMX6-lys1* (Chen et al., 2017) to get pUC119-*Padh81*-*SNF7*-GBP-mCherry(C)-hphMX6-lys1*. This plasmid was linearized with XbaI on *SNF7* and integrated to target strains.

Antibodies used in this study were mouse anti-GFP (sc-9996; Santa Cruz Biotechnology), rabbit anti-Ape1 (gift of Y. Ohsumi, Tokyo Institute of Technology, Tokyo, Japan), mouse anti-GST (EBA-001-100ul; UBio Biotechnology), mouse anti-HA (sc-7392; Santa Cruz Biotechnology), rabbit anti-glucose-6-phosphate dehydrogenase (G6PDH, A9521; Sigma-Aldrich), HRP-linked goat anti-mouse IgG (GAM0072; Multisciences), and HRP-linked goat anti-rabbit (GAR0072; Multisciences).

### Yeast culture conditions and induction of autophagy

For live-cell fluorescence microscopy and biochemical analysis, overnight cultured cells in YPD or selective medium were inoculated at ∼0.03 OD_600_ to grow ∼8 h to mid-log phase at 26°C in YPD or selective medium, reinoculated at ∼0.05 OD_600_ to reach ∼0.5 OD_600_. Autophagy was induced by adding 10 nM rapamycin to the culture in YPD for 4 h at 26°C or by shifting cells from YPD to SD-N (without amino acid and ammonium sulfate) medium for 30 min to 2 h of starvation at 26°C as indicated. For staining the vacuole or class E compartments, 1.6 µM of FM4-64 was added to the medium 1 h before harvesting cells.

### Immunoblot analysis

Immunoblot analyses were performed with indicated antibodies, subjected to ECL HRP substrate (P90720; Millipore) and exposed to x-ray film (XBT; Carestream) to generate image with an HQ-350XT film developer (Huqiu Imaging Technology). Immunoblots were quantified with ImageJ software (National Institutes of Health) for band density. G6PDH was used as a loading control for quantification.

### Live-cell microscopy and time-lapse imaging

Cells expressing fluorescent proteins or stained by FM4-64 or Alexa Fluor conjugated antibody were examined by one of the following fluorescence microscopies: Nikon inverted research microscope Eclipse Ti (Nikon) equipped with a 100 × 1.3-NA oil-immersion objective lens, digital camera (DS-Fi, high-density CCD; Nikon), and acquisition software NIS Elements FW; an UltraVIEW spinning-disk confocal scanner unit (PerkinElmer) equipped with Nikon A1 microscopy carrying Nikon Apochromat TIRF 100 × 1.49-NA oil-immersion objective lens, a digital camera (C9100-23B EMCCD; Hamamatsu), and acquisition software Volocity 6.3.0; or a Leica confocal microscope TCS SP8 (Leica Microsystems) equipped with high-contrast Plan Apochromat 100 × 1.4-NA oil-immersion objective lens, photomultiplier tube detectors and Hybrid detectors, and acquisition software Leica LAS X. When confocal microscopes were used, a Z-stack of 13 stacks of 0.3-µm step size was applied. More than five fields for each sample were visualized in the indicated growth medium at room temperature with two or three channels (488 nm for GFP/NG and 561 for mCherry/FM4-64 or 405 for BFP). Images were processed for brightness and contrast with Photoshop CS6 (Adobe) and assembled with Illustrator CS5 (Adobe) for publication. Insets in Fig. 2 D, Fig. 5 (A and C), Fig. 6 (A–C), Fig. 10 A, and Fig. S6 B show 2.5–4× magnification of the framed area. For time-lapse imaging, cells in mid-log phase were shifted to SD-N for 15 min to induce autophagy, and then spotted on SD-N with 2% agar mounted on glass slides. Images were captured at a fixed rate of 6 s/time point with two channels (488 nm for GFP and 561 nm for mCherry) and three Z-stacks (step size of 0.6 µm) using UltraVIEW spinning-disk confocal scanner unit for 25–40 min.

### Transmission electron microscopy

Cells were grown, processed, and quantified for transmission EM as previously described (Zhou et al., 2017).

### Autophagy assays

Autophagy cargo processing by immunoblot analysis was done as previously described using anti-GFP and anti-Ape1 antibodies to detect processing of GFP-Atg8 and Ape1, respectively (Zhou et al., 2017). For the Pho8Δ60 alkaline phosphatase assay, one half of mid-log phase cells cultured in YPD medium at 26°C was shifted to SD-N for starvation for 4 h, and the other half was further growing in YPD for 1.5 h. Proteins were extracted and alkaline phosphatase activity was measured as previously described (Noda and Klionsky, 2008). Cell viability during nitrogen starvation was determined using cell growth on YPD (Lipatova et al., 2012) or trypan blue staining (Strober, 2015).

### Protease-protection assays

For the conventional protease-protection assay, cells expressing GFP-Atg8 were grown to mid-log phase in YPD medium and shifted to SD-N for 2 h for starvation. The cells were spheroplasted and lysed, unbroken cells were removed by a centrifugation at 5,000× *g* (P5) for 5 min, and membrane components were collected by a centrifugation at 10,000× *g* for 10 min to yield P10. The P10 membrane fraction was resuspended in PS200 buffer and treated with PK (140 µg/ml) and/or Triton X-100 (TX) as previously described (Nair et al., 2011). Following the PK treatment with or without TX, proteins were precipitated with TCA for immunoblot analysis using anti-GFP or anti-Ape1 antibodies. Membrane fractions from mutant cells contain undegraded cargos, prApe1, or GFP-Atg8; both cargos are completely degraded when PK is added together with TX to yield mApe1 and GFP, respectively. Cargos are protected when they are not degraded by PK without detergent. In *atg1Δ* mutant cells, APs do not form and autophagy cargos are not protected. In mutant cells that accumulate closed APs (e.g., *ypt7Δ*) or ABs inside the vacuole (e.g., *pep4Δ*), ∼50% of the cargo is protected from the protease. Mutant cells that accumulate APs as determined by microscopy, in which cargos are not protected from the protease, accumulate open APs.

In the modified microscopy-based protease-protection assay, following the PK ± TX treatment, samples were centrifuged at 10,000× *g* for 5 min, followed with removing supernatant and washing with PS200 buffer twice to remove degraded peptides. Washed samples, containing comparable amounts of DIC particles as estimated from DIC pictures, were mounted on glass slides and observed by a Leica SP8 confocal microscope for DIC and GFP-Atg8. GFP-Atg8 dots are seen in membrane fractions from all cells; GFP-Atg8 is almost completely degraded when PK is added together with detergent (TX); protease protection of GFP-Atg8 is determined when PK is added to membranes without detergent.

### Anti-cargo antibodies accessibility assay

P10 membrane fractions purified from GFP-Atg8–expressing cells as described for the protease-protection assays (above) were mounted on coverslips using 0.1% poly-l-lysine. Samples were infiltrated in PBS buffer (1 ml) with 10 mg/ml BSA for 15 min blocking at room temperature. Samples incubated with anti-Ape1 primary antibody (1:1,000) overnight and with anti-rabbit IgG H&L as a secondary antibody (Alexa Fluo 647; 1:5,000) for 4 h. Samples were washed four times with PBS buffer with 10 mg/ml BSA (0.8 ml) after each antibody treatment and were examined by a Leica SP8 confocal microscope with two channels of 488 nm and 633 nm to visualize GFP-Atg8 and Alexa Fluo 647–marked Ape1, respectively. In mutant cells that accumulate closed APs (e.g., *ypt7Δ*) or ABs inside the vacuole (e.g., *pep4Δ*) <10% of the Atg8 dots colocalized with the anti-Ape1 antibodies. Single and double ESCRT mutants *snf7Δ* and *vps4Δ* accumulate APs (Figs. 2 and 3), but ∼40% of the Atg8 dots colocalize with anti-Ape1 antibodies, indicating that these APs are open.

### Biochemical complementation using the protease-protection assay

Recombinant proteins were purified from bacteria expressing GST-tagged Snf7, Vps4^wt^, or Vps4^K179A^ using affinity purification followed by thrombin digestion to remove the proteins from the tags as previously described (Jones et al., 1995). Snf7 and Vps4 proteins were incubated with P10 membrane fractions prepared from *snf7Δ* or *vps4Δ* mutant cells, respectively. Specifically, P10 was resuspended in 100 μl reaction buffer (100 mM KOAc, 20 mM Hepes, pH 7.4, and 5 mM MgOAc) with 1 mM sorbitol and incubated at 26°C for 5 min with 600 nM recombinant Snf7 (Wollert et al., 2009) or with 100 nM recombinant Vps4 or Vps4^K179A^ (Adell et al., 2014). ATP regeneration system (10 mM phosphocreatine, 20 U/ml creatine phosphokinase, and 0.8 mM ATP) was added to reactions with recombinant Vps4 or Vps4^K179A^ proteins. After the incubation, the samples were subjected to protease-protection assay and immunoblotting analysis as described above (*ypt7Δ* was used as a control for the protease-protection assays).

### ATP hydrolysis assay

Recombinant Vps4 and Vps4^K179A^ proteins purified as described above were dialyzed in the ATPase buffer (100 mM KOAc, 20 mM Hepes, pH 7.4, 5 mM MgOAc, 1 mM sorbitol) and stored at −80°C. Their ATPase activity was determined using a colorimetric assay as previously described (Rule et al., 2016). Briefly, Vps4 proteins (4 µM) were incubated in ATPase buffer at 35°C. The reaction was started with the addition of 2 mM ATP (final concentration) to a final reaction volume of 50 µl and proceeded for 30 min. At indicated time points, samples were removed and frozen at −80°C. Detection of phosphate (PO_4_^−^) with malachite green reagent (Biomol Green, BML-AK111; Enzo Life Sciences) was performed as recommended by the manufacturer. The rate of ATPase hydrolysis was calculated as PO_4_^−^ per micromole of protein. The data are presented as the mean ± SEM of three repeats.

### BiFC assay

Vectors, pVC with a pUG34 backbone expressing the C-terminal half of Venus, and pVN with a pUG36 backbone expressing the N-terminal half of Venus, were used in BiFC assay (Gong et al., 2013). Cells cotransformed with pVC-*SNF7* and pVN-*ATGX* were grown in SD-Ura-His selection medium at 26°C to mid-log phase and induced for protein expression by shifting the cells to an SD-Ura-His-Met medium for 1.5 h. Cells were further starved in SD-N for 30 min and examined by a Nikon inverted research microscope Eclipse Ti.

### GST pulldown assay

Protein interaction between Snf7 and Atg17 was confirmed with GST pulldown. One protein was fused to pYEX4T-1 (GST) and the other was endogenously labeled with 3×HA tag in yeast cells. Cells coexpressing the two proteins were grown in SD-Ura-Leu medium overnight at 26°C to mid-log phase. After 2 h induction of the GST fused protein, by adding 500 µM CuSO_4_, cells were further starved in SD-N for 30 min. Cells were then collected and subjected to GST pulldown assay as previously described (Morozova et al., 2006). The GST-tagged pellets were analyzed by immunoblotting using mouse anti-GST to assess the GST-tagged protein, mouse anti-HA to assess the coprecipitation of the HA-tagged protein, and rabbit anti-G6PDH to probe for endogenous G6PDH as a loading control.

### Statistical analyses

IBM SPSS Statistics was used for determining statistical significance for experiments with three or more repeats using ANOVA as previously described (Zhou et al., 2017). The paired two-tailed Student’s *t* test analysis was applied for determining statistical significance for experiments with two repeats or for a pair of samples, which are not applicable to ANOVA analysis.

### Online supplemental material

Fig. S1 shows that deletion of genes encoding representative subunits from each of the ESCRT complexes causes defects in starvation-induced autophagy. Fig. S2 shows that deletion of genes encoding representative subunits from each of the ESCRT complexes results in Atg8 cluster accumulation. Fig. S3 shows genetic complementation of *vps4Δ* and *snf7Δ* autophagy phenotypes by overexpression of their cognate protein. Fig. S4 shows genetic complementation of AP cluster and class E compartment accumulation in *vps4Δ* and *snf7Δ* mutant cells by expressing their cognate protein. Fig. S5 shows that recombinant and tagged ESCRT subunits are functional. Fig. S6 shows the Rab5 GTPase Vps21 and the ESCRT subunit Vps4 function in the same autophagy pathway. Fig. S7 shows a characterization of the Atg–Snf7 BiFC interaction. Video 1 shows the dynamic nature of GFP-Atg8 in WT cells. Video 2 shows the dynamic nature of GFP-Atg8 clusters in *vps4Δ* mutant cells. Video 3 shows dynamic colocalization of Snf7-mCherry and GFP-Atg8 in WT cells. Video 4 shows that dynamic colocalization of Snf7-mCherry and GFP-Atg8 is dependent on Vps21. Table S1 presents yeast strains, plasmids, and oligos used in this study.

## Supplementary Material

Supplemental Materials (PDF)

Table S1 (PDF)

Video 1

Video 2

Video 3

Video 4

## References

[bib1] Adell M.A., Vogel G.F., Pakdel M., Müller M., Lindner H., Hess M.W., and Teis D. 2014 Coordinated binding of Vps4 to ESCRT-III drives membrane neck constriction during MVB vesicle formation. J. Cell Biol. 205:33–49. 10.1083/jcb.201310114 24711499 PMC3987140

[bib2] Alfred V., and Vaccari T. 2016 When membranes need an ESCRT: Endosomal sorting and membrane remodelling in health and disease. Swiss Med. Wkly. 146:w14347. 27631343 10.4414/smw.2016.14347

[bib3] Alonso Y Adell M., Migliano S.M., and Teis D. 2016 ESCRT-III and Vps4: A dynamic multipurpose tool for membrane budding and scission. FEBS J. 283:3288–3302. 10.1111/febs.13688 26910595

[bib4] Babst M., Sato T.K., Banta L.M., and Emr S.D. 1997 Endosomal transport function in yeast requires a novel AAA-type ATPase, Vps4p. EMBO J. 16:1820–1831. 10.1093/emboj/16.8.1820 9155008 PMC1169785

[bib5] Chen Y., Zhou F., Zou S., Yu S., Li S., Li D., Song J., Li H., He Z., Hu B., 2014 A Vps21 endocytic module regulates autophagy. Mol. Biol. Cell. 25:3166–3177. 10.1091/mbc.e14-04-0917 25143401 PMC4196867

[bib6] Chen Y.H., Wang G.Y., Hao H.C., Chao C.J., Wang Y., and Jin Q.W. 2017 Facile manipulation of protein localization in fission yeast through binding of GFP-binding protein to GFP. J. Cell Sci. 130:1003–1015. 10.1242/jcs.198457 28082423

[bib7] Choi A.M., Ryter S.W., and Levine B. 2013 Autophagy in human health and disease. N. Engl. J. Med. 368:651–662. 10.1056/NEJMra1205406 23406030

[bib8] Christ L., Raiborg C., Wenzel E.M., Campsteijn C., and Stenmark H. 2017 Cellular functions and molecular mechanisms of the ESCRT membrane-scission machinery. Trends Biochem. Sci. 42:42–56. 10.1016/j.tibs.2016.08.016 27669649

[bib9] Coonrod E.M., and Stevens T.H. 2010 The yeast vps class E mutants: The beginning of the molecular genetic analysis of multivesicular body biogenesis. Mol. Biol. Cell. 21:4057–4060. 10.1091/mbc.e09-07-0603 21115849 PMC2993735

[bib10] Davies C.W., Stjepanovic G., and Hurley J.H. 2015 How the Atg1 complex assembles to initiate autophagy. Autophagy. 11:185–186. 10.4161/15548627.2014.984281 25700739 PMC4502730

[bib11] Dikic I., and Elazar Z. 2018 Mechanism and medical implications of mammalian autophagy. Nat. Rev. Mol. Cell Biol. 19:349–364. 10.1038/s41580-018-0003-4 29618831

[bib12] Djeddi A., Michelet X., Culetto E., Alberti A., Barois N., and Legouis R. 2012 Induction of autophagy in ESCRT mutants is an adaptive response for cell survival in C. elegans. J. Cell Sci. 125:685–694. 10.1242/jcs.091702 22389403

[bib13] Feng Y., He D., Yao Z., and Klionsky D.J. 2014 The machinery of macroautophagy. Cell Res. 24:24–41. 10.1038/cr.2013.168 24366339 PMC3879710

[bib14] Filimonenko M., Stuffers S., Raiborg C., Yamamoto A., Malerød L., Fisher E.M., Isaacs A., Brech A., Stenmark H., and Simonsen A. 2007 Functional multivesicular bodies are required for autophagic clearance of protein aggregates associated with neurodegenerative disease. J. Cell Biol. 179:485–500. 10.1083/jcb.200702115 17984323 PMC2064794

[bib15] Galluzzi L., Baehrecke E.H., Ballabio A., Boya P., Bravo-San Pedro J.M., Cecconi F., Choi A.M., Chu C.T., Codogno P., Colombo M.I., 2017 Molecular definitions of autophagy and related processes. EMBO J. 36:1811–1836. 10.15252/embj.201796697 28596378 PMC5494474

[bib16] Gong T., Liao Y., He F., Yang Y., Yang D.D., Chen X.D., and Gao X.D. 2013 Control of polarized growth by the Rho family GTPase Rho4 in budding yeast: Requirement of the N-terminal extension of Rho4 and regulation by the Rho GTPase-activating protein Bem2. Eukaryot. Cell. 12:368–377. 10.1128/EC.00277-12 23264647 PMC3571307

[bib17] Hammerling B.C., Najor R.H., Cortez M.Q., Shires S.E., Leon L.J., Gonzalez E.R., Boassa D., Phan S., Thor A., Jimenez R.E., 2017 A Rab5 endosomal pathway mediates Parkin-dependent mitochondrial clearance. Nat. Commun. 8:14050 10.1038/ncomms14050 28134239 PMC5290275

[bib18] Henne W.M., Stenmark H., and Emr S.D. 2013 Molecular mechanisms of the membrane sculpting ESCRT pathway. Cold Spring Harb. Perspect. Biol. 5:a016766 10.1101/cshperspect.a016766 24003212 PMC3753708

[bib19] Hurley J.H., and Hanson P.I. 2010 Membrane budding and scission by the ESCRT machinery: it’s all in the neck. Nat. Rev. Mol. Cell Biol. 11:556–566. 10.1038/nrm2937 20588296 PMC2922035

[bib20] Jones S., Litt R.J., Richardson C.J., and Segev N. 1995 Requirement of nucleotide exchange factor for Ypt1 GTPase mediated protein transport. J. Cell Biol. 130:1051–1061. 10.1083/jcb.130.5.1051 7657691 PMC2120555

[bib21] Klionsky D.J., and Eskelinen E.L. 2014 The vacuole versus the lysosome: When size matters. Autophagy. 10:185–187. 10.4161/auto.27367 24343261 PMC5396098

[bib22] Lefebvre C., Legouis R., and Culetto E. 2018 ESCRT and autophagies: Endosomal functions and beyond. Semin. Cell Dev. Biol. 74:21–28. 10.1016/j.semcdb.2017.08.014 28807884

[bib23] Liang Y., Morozova N., Tokarev A.A., Mulholland J.W., and Segev N. 2007 The role of Trs65 in the Ypt/Rab guanine nucleotide exchange factor function of the TRAPP II complex. Mol. Biol. Cell. 18:2533–2541. 10.1091/mbc.e07-03-0221 17475775 PMC1924837

[bib24] Lipatova Z., Belogortseva N., Zhang X.Q., Kim J., Taussig D., and Segev N. 2012 Regulation of selective autophagy onset by a Ypt/Rab GTPase module. Proc. Natl. Acad. Sci. USA. 109:6981–6986. 10.1073/pnas.1121299109 22509044 PMC3344974

[bib25] Liu X.M., Sun L.L., Hu W., Ding Y.H., Dong M.Q., and Du L.L. 2015 ESCRTs cooperate with a selective autophagy receptor to mediate vacuolar targeting of soluble cargos. Mol. Cell. 59:1035–1042. 10.1016/j.molcel.2015.07.034 26365378

[bib26] Liu X., Mao K., Yu A.Y.H., Omairi-Nasser A., Austin J. II, Glick B.S., Yip C.K., and Klionsky D.J. 2016 The Atg17-Atg31-Atg29 complex coordinates with Atg11 to recruit the Vam7 SNARE and mediate autophagosome-vacuole fusion. Curr. Biol. 26:150–160. 10.1016/j.cub.2015.11.054 26774783 PMC4729596

[bib27] Mercer T.J., Gubas A., and Tooze S.A. 2018 A molecular perspective of mammalian autophagosome biogenesis. J. Biol. Chem. 293:5386–5395. 10.1074/jbc.R117.810366 29371398 PMC5900756

[bib28] Morozova N., Liang Y., Tokarev A.A., Chen S.H., Cox R., Andrejic J., Lipatova Z., Sciorra V.A., Emr S.D., and Segev N. 2006 TRAPPII subunits are required for the specificity switch of a Ypt-Rab GEF. Nat. Cell Biol. 8:1263–1269. 10.1038/ncb1489 17041589

[bib29] Mukherjee A., Patel B., Koga H., Cuervo A.M., and Jenny A. 2016 Selective endosomal microautophagy is starvation-inducible in Drosophila. Autophagy. 12:1984–1999. 10.1080/15548627.2016.1208887 27487474 PMC5103356

[bib30] Müller M., Schmidt O., Angelova M., Faserl K., Weys S., Kremser L., Pfaffenwimmer T., Dalik T., Kraft C., Trajanoski Z., 2015 The coordinated action of the MVB pathway and autophagy ensures cell survival during starvation. eLife. 4:e07736 10.7554/eLife.07736 25902403 PMC4424281

[bib31] Nair U., Thumm M., Klionsky D.J., and Krick R. 2011 GFP-Atg8 protease protection as a tool to monitor autophagosome biogenesis. Autophagy. 7:1546–1550. 10.4161/auto.7.12.18424 22108003 PMC3327617

[bib32] Nakatogawa H., Suzuki K., Kamada Y., and Ohsumi Y. 2009 Dynamics and diversity in autophagy mechanisms: Lessons from yeast. Nat. Rev. Mol. Cell Biol. 10:458–467. 10.1038/nrm2708 19491929

[bib33] Nara A., Mizushima N., Yamamoto A., Kabeya Y., Ohsumi Y., and Yoshimori T. 2002 SKD1 AAA ATPase-dependent endosomal transport is involved in autolysosome formation. Cell Struct. Funct. 27:29–37. 10.1247/csf.27.29 11937716

[bib34] Noda T., and Klionsky D.J. 2008 The quantitative Pho8Delta60 assay of nonspecific autophagy. Methods Enzymol. 451:33–42. 10.1016/S0076-6879(08)03203-5 19185711

[bib35] Numrich J., and Ungermann C. 2014 Endocytic Rabs in membrane trafficking and signaling. Biol. Chem. 395:327–333. 10.1515/hsz-2013-0258 24158421

[bib36] Ohsumi Y. 2014 Historical landmarks of autophagy research. Cell Res. 24:9–23. 10.1038/cr.2013.169 24366340 PMC3879711

[bib37] Oku M., and Sakai Y. 2018 Three distinct types of microautophagy based on membrane dynamics and molecular machineries. BioEssays. 40:e1800008 10.1002/bies.201800008 29708272

[bib38] Oku M., Maeda Y., Kagohashi Y., Kondo T., Yamada M., Fujimoto T., and Sakai Y. 2017 Evidence for ESCRT- and clathrin-dependent microautophagy. J. Cell Biol. 216:3263–3274. 10.1083/jcb.201611029 28838958 PMC5626533

[bib39] Reggiori F., and Ungermann C. 2017 Autophagosome maturation and fusion. J. Mol. Biol. 429:486–496. 10.1016/j.jmb.2017.01.002 28077293

[bib40] Rieder S.E., Banta L.M., Köhrer K., McCaffery J.M., and Emr S.D. 1996 Multilamellar endosome-like compartment accumulates in the yeast vps28 vacuolar protein sorting mutant. Mol. Biol. Cell. 7:985–999. 10.1091/mbc.7.6.985 8817003 PMC275948

[bib41] Rothbauer U., Zolghadr K., Muyldermans S., Schepers A., Cardoso M.C., and Leonhardt H. 2008 A versatile nanotrap for biochemical and functional studies with fluorescent fusion proteins. Mol. Cell. Proteomics. 7:282–289. 10.1074/mcp.M700342-MCP200 17951627

[bib42] Rubinsztein D.C., Codogno P., and Levine B. 2012 Autophagy modulation as a potential therapeutic target for diverse diseases. Nat. Rev. Drug Discov. 11:709–730. 10.1038/nrd3802 22935804 PMC3518431

[bib43] Rule C.S., Patrick M., and Sandkvist M. 2016 Measuring in vitro ATPase activity for enzymatic characterization. J. Vis. Exp. 114:e54305 10.3791/54305 PMC5091952 27584824

[bib44] Russell M.R., Shideler T., Nickerson D.P., West M., and Odorizzi G. 2012 Class E compartments form in response to ESCRT dysfunction in yeast due to hyperactivity of the Vps21 Rab GTPase. J. Cell Sci. 125:5208–5220. 10.1242/jcs.111310 22899724 PMC3533395

[bib45] Rusten T.E., Vaccari T., Lindmo K., Rodahl L.M., Nezis I.P., Sem-Jacobsen C., Wendler F., Vincent J.P., Brech A., Bilder D., and Stenmark H. 2007 ESCRTs and Fab1 regulate distinct steps of autophagy. Curr. Biol. 17:1817–1825. 10.1016/j.cub.2007.09.032 17935992

[bib46] Sahu R., Kaushik S., Clement C.C., Cannizzo E.S., Scharf B., Follenzi A., Potolicchio I., Nieves E., Cuervo A.M., and Santambrogio L. 2011 Microautophagy of cytosolic proteins by late endosomes. Dev. Cell. 20:131–139. 10.1016/j.devcel.2010.12.003 21238931 PMC3025279

[bib47] Schuh A.L., and Audhya A. 2014 The ESCRT machinery: From the plasma membrane to endosomes and back again. Crit. Rev. Biochem. Mol. Biol. 49:242–261. 10.3109/10409238.2014.881777 24456136 PMC4381963

[bib48] Strober W. 2015 Trypan blue exclusion test of cell viability. Curr. Protoc. Immunol. 111:A3.B.1–3. 26529666 10.1002/0471142735.ima03bs111 PMC6716531

[bib49] Takahashi Y., He H., Tang Z., Hattori T., Liu Y., Young M.M., Serfass J.M., Chen L., Gebru M., Chen C., 2018 An autophagy assay reveals the ESCRT-III component CHMP2A as a regulator of phagophore closure. Nat. Commun. 9:2855 10.1038/s41467-018-05254-w 30030437 PMC6054611

[bib50] Tang S., Henne W.M., Borbat P.P., Buchkovich N.J., Freed J.H., Mao Y., Fromme J.C., and Emr S.D. 2015 Structural basis for activation, assembly and membrane binding of ESCRT-III Snf7 filaments. eLife. 4:e12548 10.7554/eLife.12548 26670543 PMC4720517

[bib51] Torggler R., Papinski D., and Kraft C. 2017 Assays to monitor autophagy in Saccharomyces cerevisiae. Cells. 6:6. 10.3390/cells6030023 PMC5617969 28703742

[bib52] Weidberg H., Shvets E., and Elazar Z. 2011 Biogenesis and cargo selectivity of autophagosomes. Annu. Rev. Biochem. 80:125–156. 10.1146/annurev-biochem-052709-094552 21548784

[bib53] Wollert T., Wunder C., Lippincott-Schwartz J., and Hurley J.H. 2009 Membrane scission by the ESCRT-III complex. Nature. 458:172–177. 10.1038/nature07836 19234443 PMC2743992

[bib54] Zerial M., and McBride H. 2001 Rab proteins as membrane organizers. Nat. Rev. Mol. Cell Biol. 2:107–117. 10.1038/35052055 11252952

[bib55] Zhou F., Zou S., Chen Y., Lipatova Z., Sun D., Zhu X., Li R., Wu Z., You W., Cong X., 2017 A Rab5 GTPase module is important for autophagosome closure. PLoS Genet. 13:e1007020 10.1371/journal.pgen.1007020 28934205 PMC5626503

