# Supplementary material for: Rab5-dependent autophagosome closure by ESCRT
Source: J Cell Biol. 2019 Apr 22;218(6):1908–27. doi: 10.1083/jcb.201811173 (PMC6548130; doi:10.1083/jcb.201811173)
Supplement: Supplemental Materials (PDF) [file JCB_201811173_sm.pdf]

Supplemental material

Zhou et al., https://doi.org/10.1083/jcb.201811173

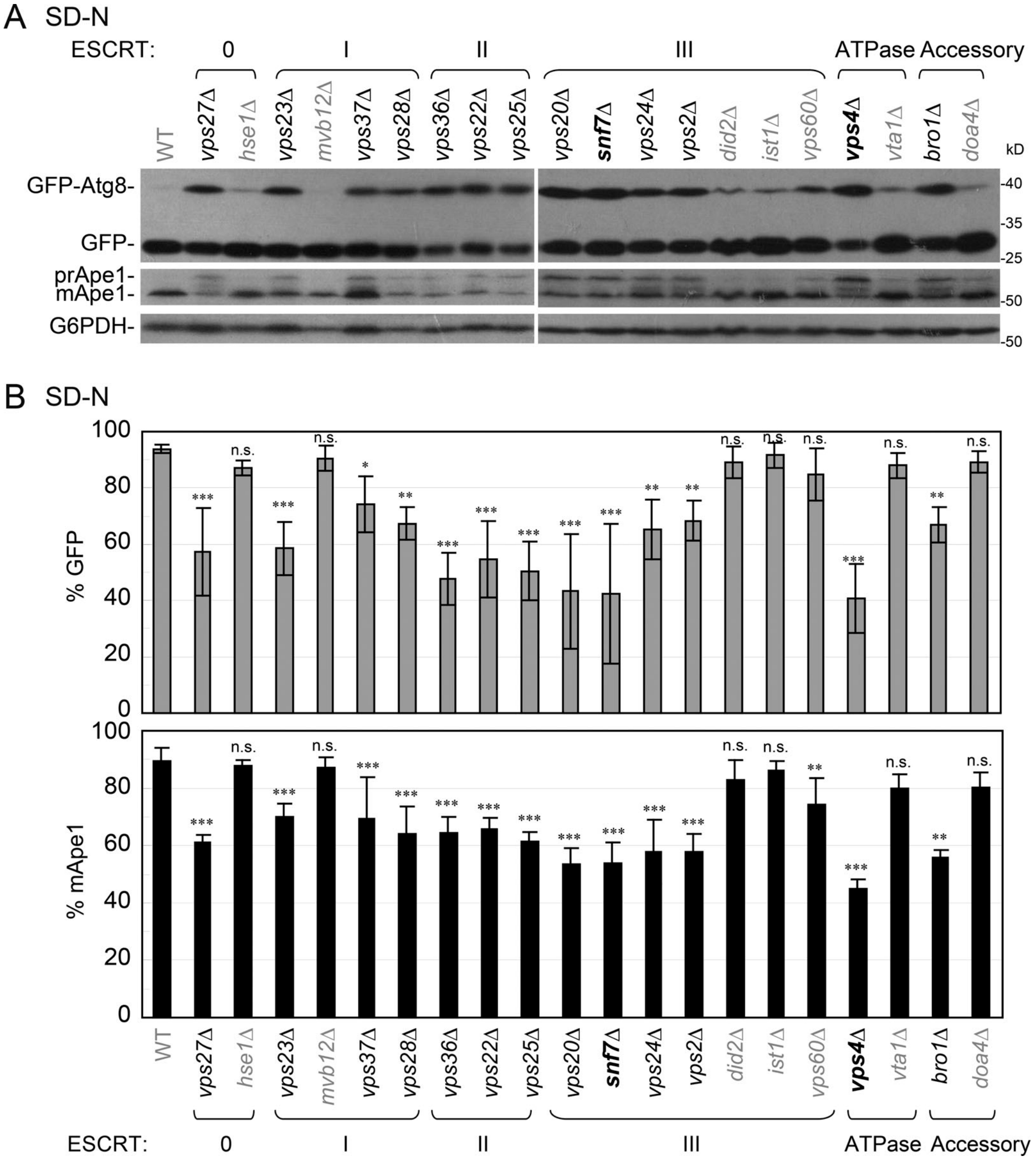

Figure S1. **Deletion of genes encoding representative subunits from each of the ESCRT complexes causes defects in starvation-induced autophagy. (A)** Immunoblot analyses of processing of two autophagy cargos, GFP-Atg8 and Ape1, in WT and mutant cells deleted for individual ESCRT subunits during starvation. Experiments were done as in Fig. 1 E. Shown from top to bottom: ESCRT complex number (0–III, or accessory factors), deletion mutant, GFP-Atg8 immunoblot, Ape1 immunoblot, and G6PDH (loading control). **(B)** The majority of ESCRT deletion mutants exhibit partial defects (40–60%) in processing of autophagy cargos. Bar graphs showing the quantification of immunoblots from A. Percentage of processed proteins, GFP (top) and mApe1 (bottom), for each mutant strain. Black font: deletion strains that exhibit an autophagy defect; bold font: *snf7Δ* and *vps4Δ*; gray font: WT and mutants with a WT-like phenotype. Columns represent mean, error bars represent SD; n.s., not significant; *P < 0.05; **P < 0.01; ***P < 0.001. Results in this figure represent three independent experiments.

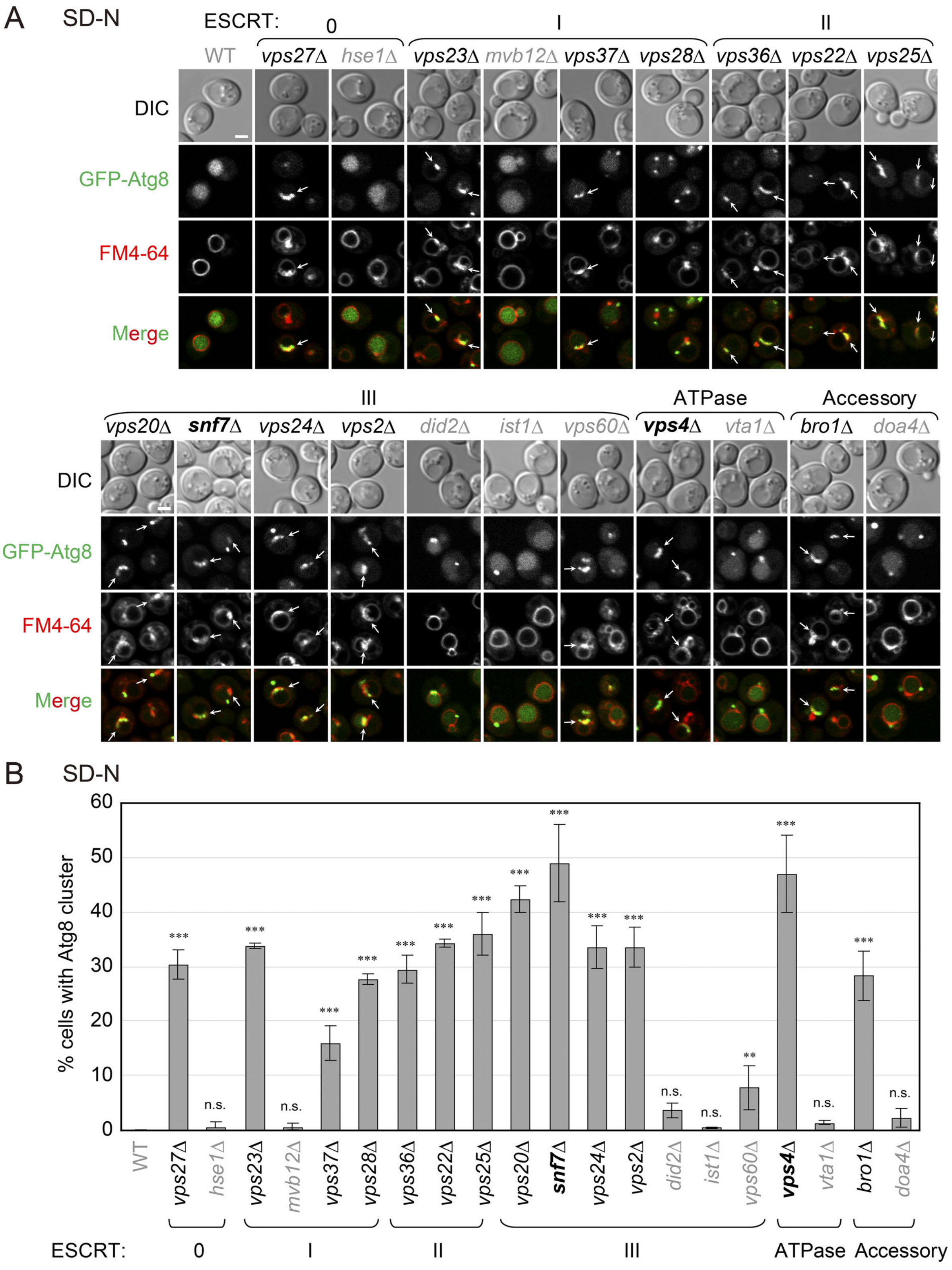

Figure S2. **Deletion of genes encoding representative subunits from each of the ESCRT complexes results in Atg8 cluster accumulation. (A)** GFP-Atg8 cluster accumulation in WT and mutant cells deleted for individual ESCRT subunits during starvation. Experiments were done as in Fig. 2 D. Shown from top to bottom: ESCRT complex number (0–III, or accessory factors), deletion mutant, DIC, GFP-Atg8, FM4-64, and merge. Arrows point to Atg8 clusters. Scale bar, 2 µm. **(B)** The majority of ESCRT deletion mutant cells accumulate Atg8 clusters (in 30–50% of their cells). Bar graphs showing the quantification of live-cell fluorescence microscopy from A: Percentage of cells with GFP-Atg8 cluster in each mutant strain. Black font: deletion strains that accumulate Atg8 clusters; bold font: *snf7Δ* and *vps4Δ*; gray font: WT and mutants with WT-like phenotype. For each strain, >900 cells were examined. Columns represent mean, error bars represent SD; n.s., not significant; **P < 0.01; ***P < 0.001. Results in this figure represent three independent experiments.

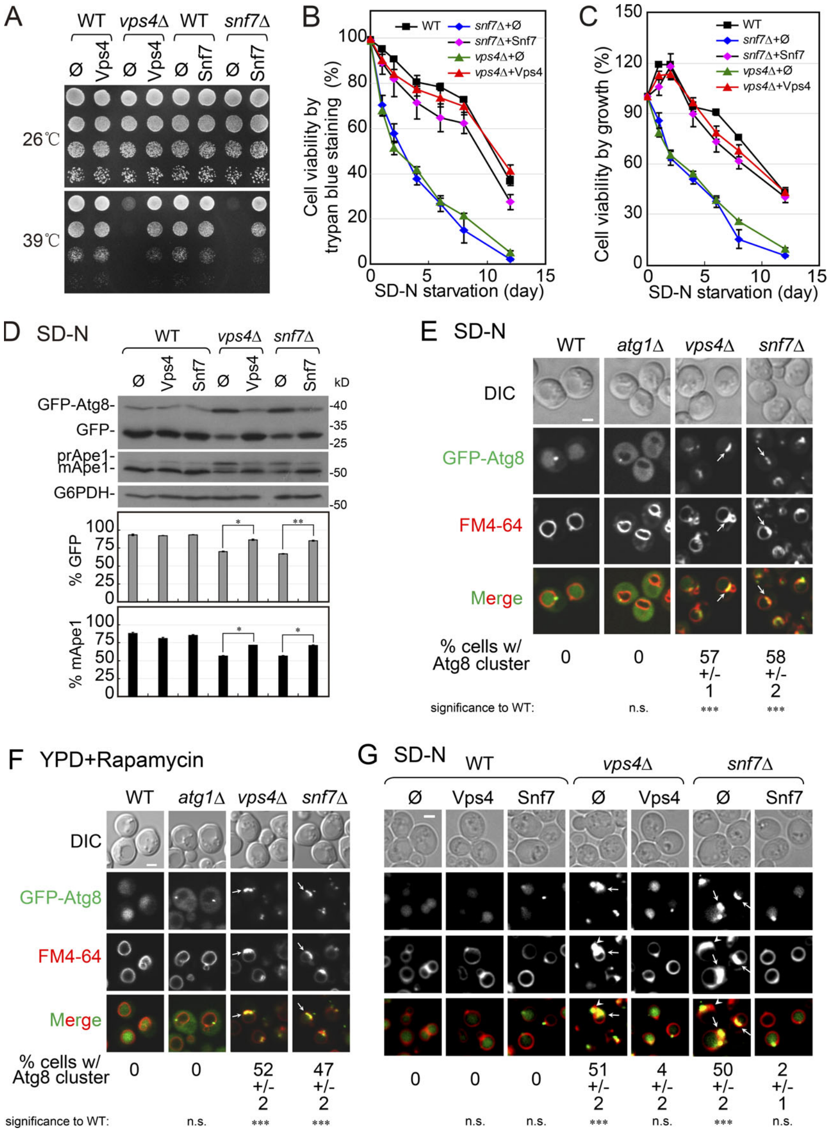

Figure S3. **Genetic complementation of *vps4Δ* and *snf7Δ* autophagy phenotypes by overexpression of their cognate protein. (A)** Overexpressed Vps4 and Snf7 complement the temperature sensitivity of *vps4Δ* and *snf7Δ* mutant cells, respectively. WT and mutant cells expressing GFP-Atg8 from its endogenous locus were transformed with plasmids: empty vector (pRS423, Ø), overexpressing Vps4 or Snf7. The ability of cells to grow on selective plates (SD-His) at 26°C (top) or 39°C (bottom) was recorded after 2 d. Shown from top to bottom: strain, plasmid, growth at 26°C and growth at 39°C (10-fold serial dilutions). Mutant cells transformed with empty vector grow like WT at 26°C but not at 39°C. When transformed with a plasmid for expression of their cognate protein, mutant cells grow also at 39°C (no effect was observed in WT cells). These results indicate that the overexpressed proteins are functional. **(B and C)** The cell viability defect of *snf7Δ* and *vps4Δ* mutant cells grown under nitrogen starvation is complemented by overexpression of their cognate proteins. Mutant cells were transformed with plasmids expressing their cognate proteins (or empty plasmid as a negative control). The experiments were done as described for Fig. 1, B and C. Cell viability, shown as a percentage of cell viability at day zero, was tested by trypan blue staining (B) and ability to form colonies on YPD plates (C) at time zero and after the indicated number of days in SD-N. Whereas mutant cells transformed with an empty plasmid lose viability faster than WT cells, overexpression of their cognate proteins complements this defect. **(D)** Overexpressed Vps4 and Snf7 partially complement the autophagy cargo-processing defects of *vps4Δ* and *snf7Δ* mutant cells, respectively. Transformants from A expressing GFP-Atg8 were tested for processing of two autophagy cargos, GFP-Atg8 (top) and Ape1 (bottom) during starvation. Experiments were done as in Fig. 1 E. Shown from top to bottom: strain, plasmid, GFP blot, Ape1 blot, bar graphs of percentage of processed cargos, GFP, and mApe1. A significant, albeit partial, suppression of the cargo-processing defects is observed when the cognate protein was expressed in mutant cells (no effect was observed in WT cells). **(E and F)** The majority of *vps4Δ* and *snf7Δ* mutant cells accumulate GFP-Atg8 clusters when autophagy is induced by nitrogen starvation (E) or rapamycin (F). Strains from left to right: WT, *atg1Δ*, *vps4Δ*, and *snf7Δ*. Cells were grown to mid-log phase in YPD and autophagy was induced by starvation (SD-N for 2 h; E) or addition of rapamycin (10 ng/ml for 4 h; F). The experiments were done as in Fig. 2 D; percentage of cells with Atg8 clusters is shown at the bottom. In WT cells Atg8 accumulates inside the vacuoles (marked with FM4-64); in *atg1Δ* mutant cells Atg8 is dispersed in the cytoplasm; in >50% of the *vps4Δ* and *snf7Δ* mutant cells Atg8 accumulates in crescent-like structures near the vacuole. **(G)** Overexpressed Vps4 and Snf7 complement the Atg8 cluster accumulation defects of *vps4Δ* and *snf7Δ* mutant cells, respectively. Autophagy was induced by starvation in transformants from A. Experiments were done as in E. Shown from top to bottom: strain, plasmid, PhC, Atg8, FM4-64, merge, and percentage of cells with an Atg8 cluster. Whereas ~50% of *vps4Δ* and *snf7Δ* mutant cells accumulate Atg8 clusters, this phenotype is seen in <4% when Vps4 and Snf7 are overexpressed, respectively (no effect was observed in WT cells). Scale bars, 2 µm. More than 600 cells were examined for the strains in E–G. Error bars and +/− in this figure represent SD; P values in D–G: n.s., not significant; $^{*}P < 0.1$; $^{**}P < 0.01$; $^{***}P < 0.001$. Results represent three independent experiments.

https://doi.org/10.1083/jcb.201811173

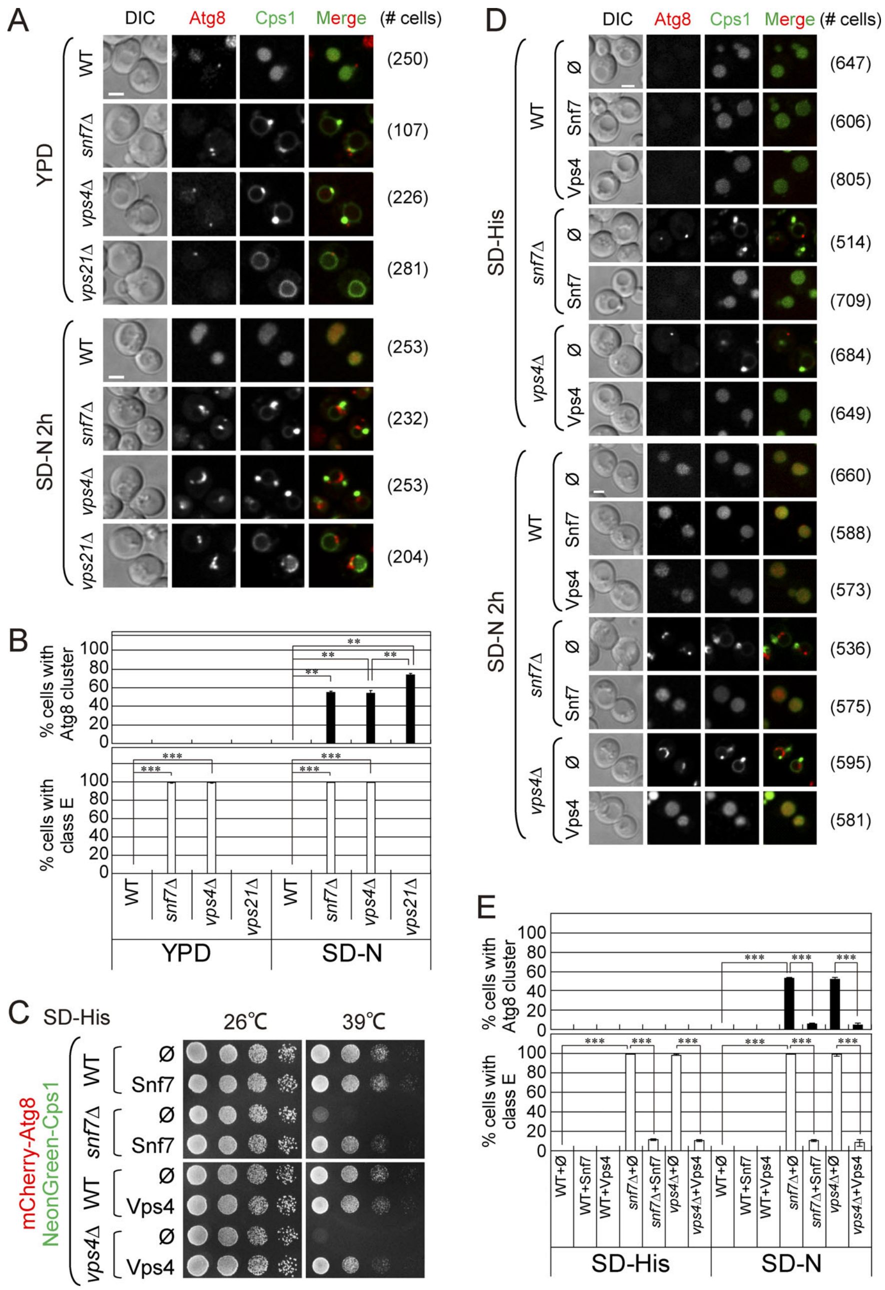

Figure S4. **Genetic complementation of AP cluster and class E compartment accumulation in *vps4Δ* and *snf7Δ* mutant cells by expressing their cognate protein. (A and B)** Accumulation of Atg8 clusters and Cps1-marked class E compartment in *vps4Δ* and *snf7Δ* mutant cells. **(A)** Cells expressing endogenously tagged mCherry-Atg8 and NG-Cps1 from a plasmid were grown in YPD (top panels) and shifted to SD-N for 2 h (bottom panels) before visualization by live-cell fluorescence microscopy. Strains from top to bottom for each growth condition: WT, *snf7Δ*, *vps4Δ*, and *vps21Δ*. Shown from left to right: growth condition, strain, DIC, Atg8, Cps1, merge, and the number of cells used for the quantification in B. Scale bar, 2 µm. While in WT cells, Atg8 and Cps1 are delivered to the vacuole during starvation; all three mutants exhibit a defect in this delivery. **(B)** Bar graphs showing quantification of data from A. Atg8 clusters accumulate during starvation in the majority of *snf7Δ*, *vps4Δ*, and *vps21Δ* during starvation (top, black bars). In contrast, Cps1-marked class E compartment accumulates in *snf7Δ* and *vps4Δ* mutant cells, but not in *vps21Δ*, during normal growth and under starvation (bottom, white bars). Columns represent mean, error bars represent SD. **(C)** Temperature growth defects of *snf7Δ* and *vps4Δ* cells expressing mCherry-Atg8 and NG-Cps1 at restrictive temperature (39°C) were complemented by Snf7 and Vps4 (overexpressed from a 2-µ plasmid), respectively. Experiments were done as in Fig. S3 A and show that the overexpressed proteins are functional. **(D and E)** Overexpressed Vps4 and Snf7 complement the accumulation defects of Atg8 cluster and class E compartment in *vps4Δ* and *snf7Δ* mutant cells, respectively. **(D)** Transformants from C were grown in media with (SD-His, top) or without nitrogen (SD-N, bottom) and analyzed by live-cell fluorescence microcopy as in A. Shown from left to right: growth condition, strain, plasmid, DIC, Atg8, Cps1, merge, and number of cells quantified in E. Scale bar, 2 µm. **(E)** Bar graphs showing quantification of data from D. Whereas ~50% of *vps4Δ* and *snf7Δ* mutant cells accumulate Atg8 clusters, this phenotype is complemented when Vps4 and Snf7 are overexpressed, respectively (no effect was observed in WT cells; top graph). Almost all *vps4Δ* and *snf7Δ* mutant cells accumulate class E compartment, and this phenotype is also complemented by expression of the cognate protein (no effect was observed in WT cells; bottom graph). Quantification and presentation methods are the same as for B. Results in this figure represent three independent experiments. **P < 0.01; ***P < 0.001.

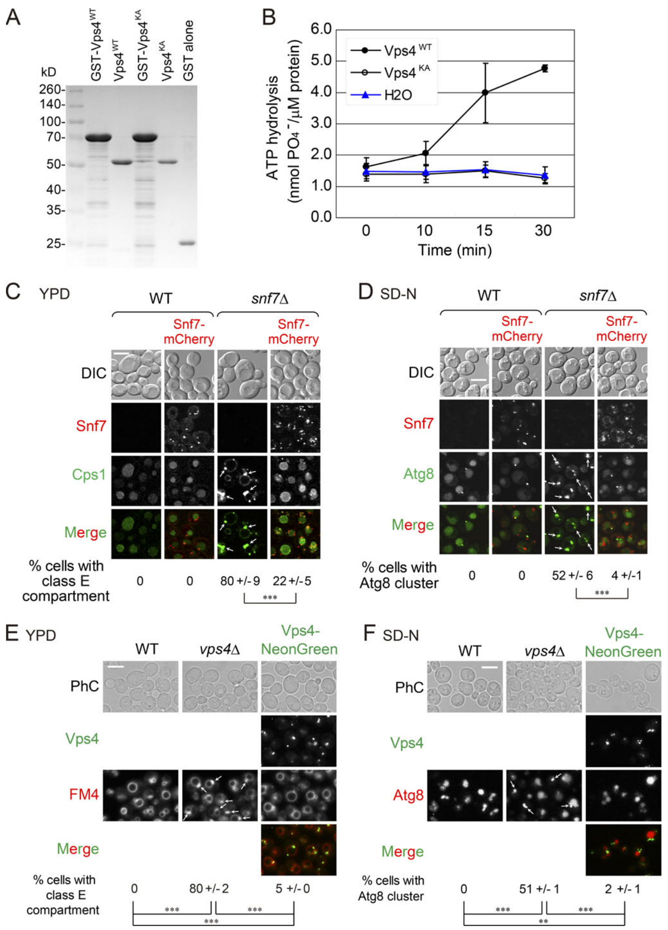

Figure S5. **Recombinant and tagged ESCRT subunits are functional. (A and B)** Recombinant protein Vps4[wt], but not the Vps4[K179A] mutant, possesses an ATPase activity. GST-tagged Vps4[WT] and Vps4[K179A] were expressed in bacteria, affinity purified using Glutathione Sepharose 4B, and then cleaved with thrombin to remove from the GST tag. **(A)** Purified proteins (5 µl) were analyzed by SDS-PAGE and Coomassie staining to assess their size and purity. Shown from left to right: molecular mass markers for GST-Vps4[wt], Vps4[wt], GST-Vps4[K179A], Vps4[K179A], and GST. **(B)** Time course of ATP hydrolysis by Vps4[WT] and Vps4[K179A] proteins (Vps4[KA]). Vps4 proteins (4 µM, or water as a negative control) were added to ATP hydrolysis reactions, and at the indicated times samples were removed to assess the production of inorganic phosphate. Graph shows accumulation of PO_4^- per µM protein at each time point. Whereas the WT protein shows an ATPase activity, the mutant protein is indistinguishable from the negative control. The data are presented as the mean ± SEM of three repeats. These proteins were used for experiments presented in Fig. 7. **(C and D)** Snf7-mCherry complements the class E compartment and AP accumulation phenotypes of *snf7Δ* mutant cells under normal growth and nitrogen starvation, respectively. **(C)** Complementation of the class E compartment accumulation phenotype of *snf7Δ* by Snf7-mCherry. Snf7 tagged with mCherry was expressed in WT and *snf7Δ* mutant cells that also express NG-Csp1. Cells were grown to mid-log phase and accumulation of Csp1-marked class E compartment was examined using live-cell fluorescence microscopy. Strains from left to right: WT, WT expressing Snf7-mCherry, *snf7Δ*, and *snf7Δ* expressing Snf7-mCherry. Shown from top to bottom: strain, DIC, mCherry, NG, merge, and percentage of cells with class E compartment (>200 cells per strain were quantified). In WT cells, regardless of the Snf7-mCherry expression, Cps1 localizes to the vacuole. In *snf7Δ* mutant cells, Cps1 clusters can be observed outside the vacuole in most cells. In *snf7Δ* mutant cells expressing Snf7-mCherry, Cps1 reaches the vacuole as in WT cells and Cps1 clusters are rare. Arrows point to Cps1 clusters. Scale bar, 5 µm. **(D)** Complementation of the AP accumulation phenotype of *snf7Δ* by Snf7-mCherry. Snf7 was tagged with mCherry in WT and *snf7Δ* mutant cells that also express GFP-Atg8. Cells were grown to mid-log phase and autophagy was induced by starvation (SD-N for 2 h). Accumulation of Atg8-marked AP clusters was examined using live-cell fluorescence microscopy. Results are shown as in C, except that at the bottom, quantification shows percentage of cells with Atg8 clusters (>200 cells per strain were quantified). In WT cells regardless of the Snf7-mCherry expression, Atg8 localizes to the vacuole. In *snf7Δ* mutant cells, Atg8 clusters can be observed outside the vacuole of ~50% of the cells. In *snf7Δ* mutant cells expressing Snf7-mCherry, Atg8 reaches the vacuole as in WT cells and Atg clusters are rare. Arrows point to Atg8 clusters. Scale bar, 5 µm. **(E and F)** Cells expressing Vps4-NG as the only copy of Vps4 do not accumulate class E compartment or AP clusters as do *vps4Δ* mutant cells under normal growth and nitrogen starvation, respectively. **(E)** Cells expressing Vps4-NG do not accumulate class E compartment. Cells expressing Vps4-NG were grown to mid-log phase, stained with FM4-64, and accumulation of FM4-64–marked class E compartment was examined using live-cell fluorescence microscopy. Strains from left to right: WT, *vps4Δ*, and WT expressing Vps4-NG. Shown from top to bottom: strain, PhC, NG, FM4-64, merge, and percentage of cells with class E compartment (>200 cells per strain were quantified). In WT cells, FM4-64 localizes to the vacuolar membrane. In *vps4Δ* mutant cells, FM4-64 clusters can be observed near the vacuole of most cells. In WT cells expressing Vps4-NG, FM4-64 reaches the vacuole as in WT cells. Arrows point to FM4-64 clusters. Scale bar, 5 µm. **(F)** Cells expressing Vps4-NG do not accumulate Atg8 clusters. Cells expressing Vps4-NG and mCherry-Atg8 were grown and tested as described for D. Strains from left to right: WT, *vps4Δ*, and WT expressing Vps4-NG. Shown from top to bottom: strain, PhC, Vps4, Atg8, merge, and percentage of cells with Atg8 cluster (>200 cells per strain were quantified). In WT cells, under nitrogen starvation Atg8 localizes to the vacuole. In *vps4Δ* mutant cells, Atg8 clusters can be observed near the vacuole of ~50% of the cells. In WT cells expressing Vps4-NG, Atg8 reaches the vacuole as in WT cells. Arrows point to Atg8 clusters. Scale bar, 5 µm. Error bars and +/− in this figure represent SD. **P < 0.01; ***P < 0.001. Results represent two to three independent experiments.

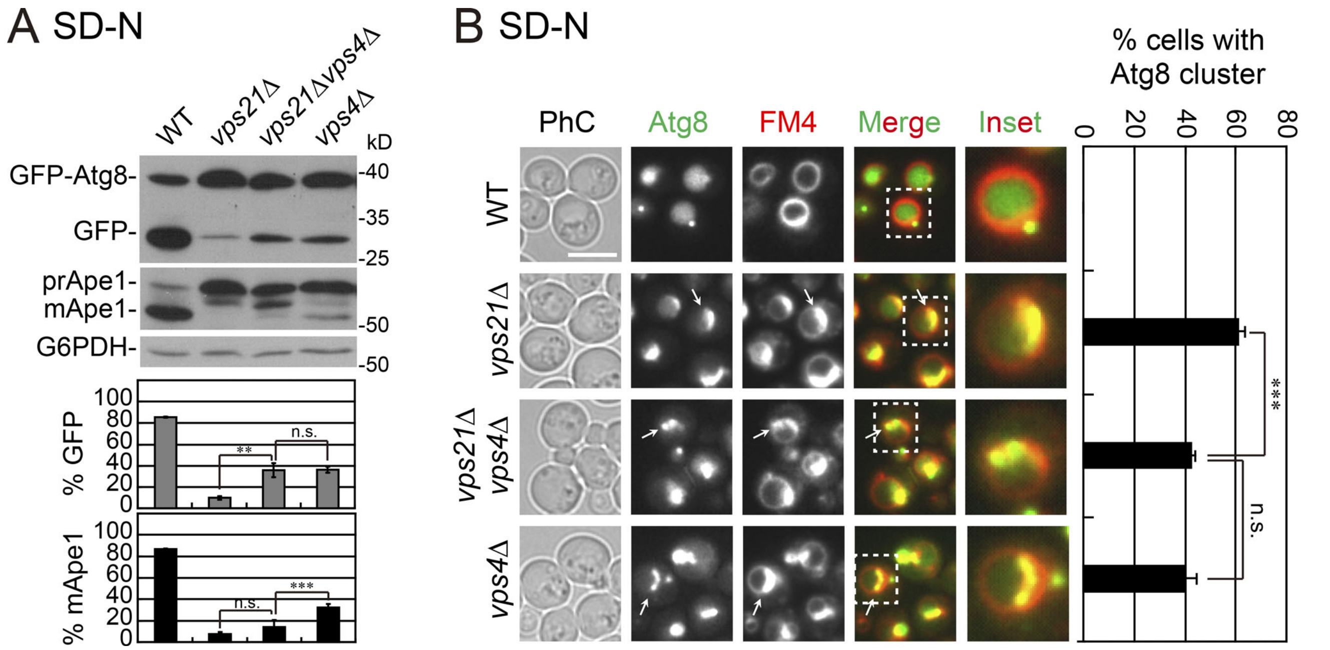

Figure S6. **The Rab5 GTPase Vps21 and the ESCRT subunit Vps4 function in the same autophagy pathway. (A)** Similar partial defects of cargo processing (GFP-Atg8 and Ape1) were observed in single and double *vps21Δ* and *vps4Δ* mutant cells during starvation-induced autophagy. Experiments were done as in Fig. 1 E. Strains from left to right: WT, *vps21Δ*, *vps21Δ vps4Δ*, and *vps4Δ*. Shown from top to bottom: strain, GFP blot, Ape1 blot, G6PDH (loading control), and bar graphs quantifying percentage of processed proteins, GFP and mApe1. **(B)** Similar Atg8 cluster accumulation defects are observed in single and double *vps21Δ* and *vps4Δ* mutant cells during starvation-induced autophagy. Experiments were done as in Fig. 2 D. Strains from top to bottom: WT, *vps21Δ*, *vps21Δ vps4Δ*, and *vps4Δ*. Shown from left to right: PhC, Atg8, FM4-64, merge, and inset (from the frame in merge). Quantification: Percentage of cells with an Atg8 cluster, is shown in the bar graph (right, >300 cells were examined for each strain). Arrows point to AP clusters. Scale bar, 5 µm. In A and B: Columns represent mean, error bars represent SD. n.s., not significant; **P < 0.01; ***P < 0.001. Results in this figure represent three independent experiments.

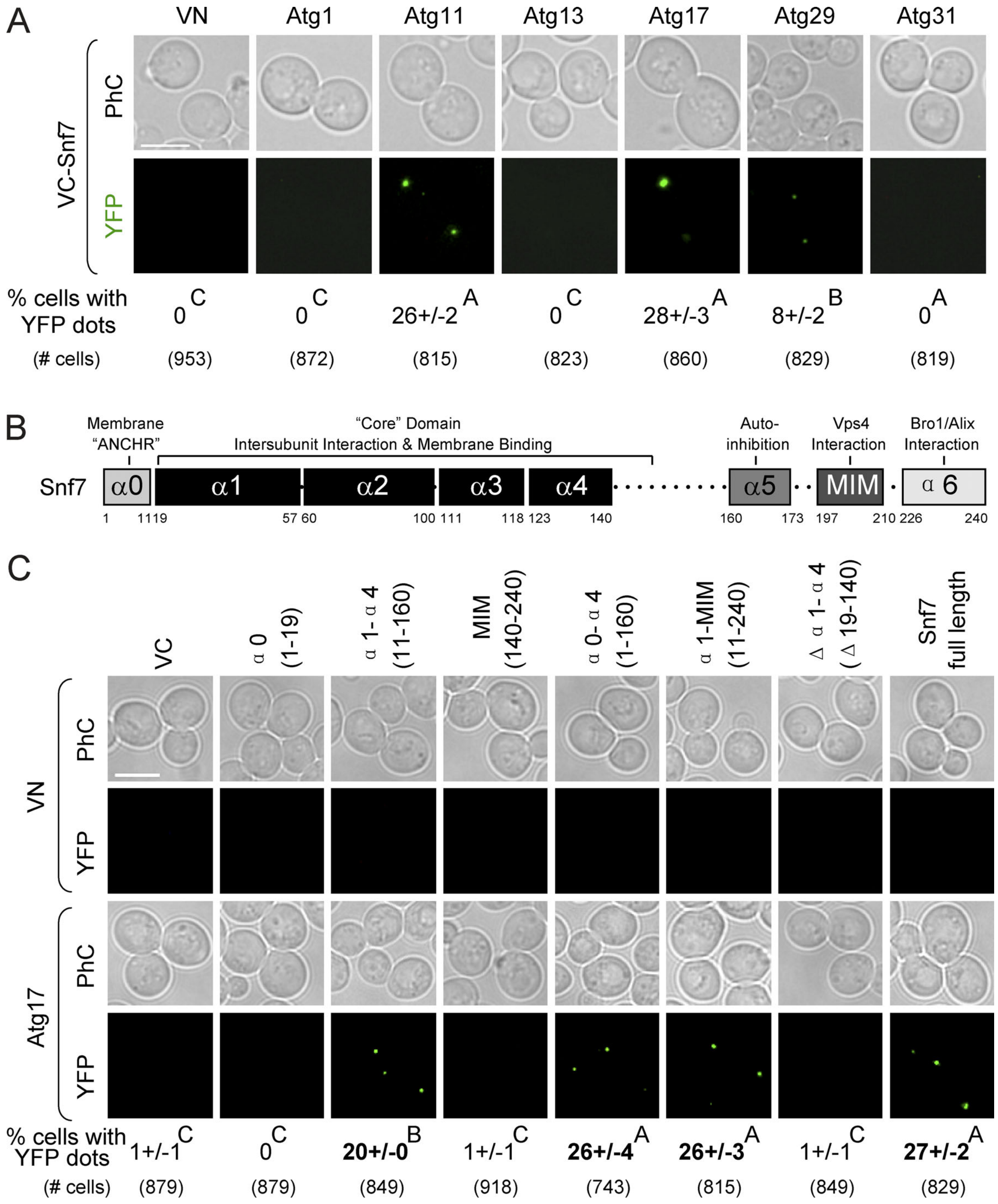

Figure S7. **Characterization of the Atg–Snf7 BiFC interaction. (A)** Snf7 interacts with Atg11 and Atg17 in the BiFC interaction assay. Cells cotransformed with VC and VN plasmids were grown as described in Materials and methods and shifted to SD-N for 30 min before YFP fluorescence was observed using live-cell fluorescence microscopy. VN plasmids shown from left to right: VN (empty plasmid); VN with the different Atgs: 1, 11, 13, 17, 29, and 31. Shown from top to bottom: PhC and YFP for cells expressing VC-Snf7, percentage of cells with YFP dots, and number of cells used for the analysis. YFP puncta showing BiFC interaction were observed only in cells expressing VC-Snf7 with VN-Atg11 or VN-Atg17 (>25% of the cells), and to a lesser extent also with VN-Atg29 (~8%). **(B and C)** The N-terminal membrane-binding domain of Snf7 is required and sufficient for its BiFC interaction with Atg17. The different domains of Snf7 (B; adapted from Tang et al., 2015) were cloned into the VC vector and cotransformed with VN (empty plasmid) or VN-Atg17. Cells were grown as described for A before YFP fluorescence was observed using live-cell fluorescence microscopy (C). Shown from left to right: VC (empty plasmid); VC with the different domains of Snf7; amino acids 1–19, 11–160, 140–240, 1–160, and 11–240; Δ of amino acids 19–140; and full-length Snf7. Shown from top to bottom: PhC and YFP for cells expressing VN (empty plasmid as a negative control), PhC and YFP for cells expressing VN-Atg17, percentage of cells with YFP dots, and number of cells used for the analysis. The minimal domains that show interaction similar to the full length (>25%) are 1–160 (26%) and 11–160 (20%). Scale bar, 2 µm. Data are presented as the mean ± SD of each variable from three independent experiments; >740 cells per strain were examined. The same capital letters of A, B, C at the right-top corner of each mean ± SD indicate no statistically significant difference, while different capital letters indicate significant difference ($P < 0.05$). Results in this figure represent three independent experiments.

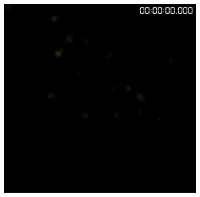

Video 1. **Time-lapse microscopy shows the dynamic nature of GFP-Atg8 in WT cells.** WT cells expressing GFP-Atg8 were grown in YPD to log phase and shifted to SD-N for 15 min before spotting them on SD-N with 2% agar for observation by time-lapse video microscopy: 6-s intervals; playing at 10 frames per second (fps). A single Atg8 punctum can be seen appearing and disappearing in a WT cell. Results shown represent three independent experiments.

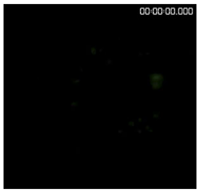

Video 2. **Time-lapse microscopy shows the dynamic nature of GFP-Atg8 clusters in *vps4Δ* mutant cells.** Mutant cells expressing GFP-Atg8 were grown in YPD to log phase and shifted to SD-N for 15 min before spotting them on SD-N with 2% agar for observation by time-lapse video microscopy: 6-s intervals; playing at 10 frames per second (fps). An individual Atg8 punctum in a *vps4Δ* mutant cell appears and stays, while additional puncta appear and individual puncta move independently. Results shown represent three independent experiments.

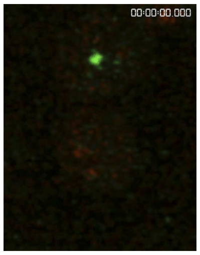

Video 3. **Dynamic colocalization of Snf7-mCherry and GFP-Atg8 in WT cells.** WT cells expressing Snf7-mCherry and GFP-Atg8 were grown in YPD medium until mid-log phase, starved in SD-N for 15 min before spotting on 2% agar with SD-N on glass slides, and monitored by time-lapse video microscopy for 25 min at 6-s intervals. Videos show Snf7-mCherry and GFP-Atg8 colocalizations for 6-s intervals, playing at 10 frames/s (fps). These data were quantified for percentage of time of colocalization and are presented in Fig. 8 D.

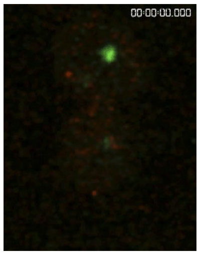

Video 4. **Dynamic colocalization of Snf7-mCherry and GFP-Atg8 is dependent on Vps21.** *vps21Δ* Mutant cells expressing Snf7-mCherry and GFP-Atg8 were grown in YPD medium until mid-log phase, starved in SD-N for 15 min before spotting on 2% agar with SD-N on glass slides, and monitored by time-lapse video microscopy for 25 min at 6-s intervals. Videos show Snf7-mCherry and GFP-Atg8 colocalizations for 6-s intervals, playing at 10 frames/s (fps). These data were quantified for percentage of time of colocalization and are presented in Fig. 8 D.

Provided online is one table as a PDF. Table S1 presents yeast strains, plasmids, and oligos used in this study.
