# Supplementary material for: Rab5-dependent autophagosome closure by ESCRT
Source: J Cell Biol. 2019 Apr 22;218(6):1908–27. doi: 10.1083/jcb.201811173 (PMC6548130; doi:10.1083/jcb.201811173)
Supplement: Table S1 (PDF) [file JCB_201811173_TableS1.pdf]

Table S1. Yeast strains, plasmids and oligos used in this study

A. Strains

Strain	Genotype	Source	Figures/Videos
YLY2422	SEY6210 *ura3::GFP-ATG8-URA3*	[1]	Fig. 1A-C, 1E-F, 2D-E, 3, S1, S2, S3, S5D, S6; Video 1
YLY5928	YLY2422 *atg1*Δ*::kanMX3*	[1]	Fig. 1A-C, 1E-F, 4, S3E-F
YLY6405	YLY2422 *snf7*Δ*::kanMX3*	This study	Fig. 1A-C, 1E-F, 3, 4A-B, 5, 7A-B, S1, S2, S3, S5D
YLY6335	YLY2422 *vps4*Δ*::kanMX3*	This study	Fig. 1A-C, 1E-F, 3, 4C-F, 5C-D, 7C-D, S1, S2, S3, S6; Video 2
YLY3359	*MATα leu2-3,112 trp1 ura3-52 pho8::pho8*Δ*60 pho13*Δ*::LEU2*	[2]	Fig. 1D
YLY3525	YLY3359 *atg1*Δ*::kanMX3*	[1]	Fig. 1D
YLY6708	YLY3359 *snf7*Δ*::kanMX3*	This study	Fig. 1D
YLY6707	YLY3359 *vps4*Δ*::kanMX3*	This study	Fig. 1D
YLY6880	YLY2422 *3×tagBFP-PHO8::LEU2*	This study	Fig. 2A-C
YLY6884	YLY6880 *snf7*Δ*::kanMX3*	This study	Fig. 2A-C
YLY6882	YLY6880 *vps4*Δ*::kanMX3*	This study	Fig. 2A-C
YLY6943	YLY6880 *atg1*Δ*::hphMX4*	This study	Fig. 2A-C
YLY6947	YLY6884 *atg1*Δ*::hphMX4*	This study	Fig. 2A-C
YLY6945	YLY6882 *atg1*Δ*::hphMX4*	This study	Fig. 2A-C
YLY3287	YLY2422 *pep4*Δ*::hphMX4*	[1]	Fig. 2D-E, 3, 4E-F, 5C-D
YLY6590	YLY6335 *pep4*Δ*::hphMX4*	This study	Fig. 2D-E, 3, 4E-F, 5C-D
YLY6919	YLY6335 *snf7*Δ*::hphMX4*	This study	Fig. 3

YLY6482	YLY6405 *ypt7Δ::hphMX4*	This study	Fig. 4A-B, 5
YLY3889	YLY2422 *ypt7Δ::hphMX4*	[1]	Fig. 4A-D, 5, 7
YLY6480	YLY6335 *ypt7Δ::hphMX4*	This study	Fig. 4C-D
YLY8500	SEY6210 *ATG8*::*mCherry-ATG8-TRP1 ATG2-GFP::kanMX3*	[3]	Fig. 6A, E
YLY8394	YLY8500 *vps21Δ::hphMX4*	This study	Fig. 6A, E
YLY2154	YLY8500 *snf7Δ::hphMX4*	This study	Fig. 6A, E
YLY2155	YLY8500 *vps4Δ::hphMX4*	This study	Fig. 6A, E
YLY9022	YLY8500 *ypt7Δ::hphMX4*	[3]	Fig. 6A, E
YLY8442	SEY6210 *ATG8*::*mCherry-ATG8-TRP1 ATG5-GFP::kanMX3*	[3]	Fig. 6B, E
YLY8473	YLY8442 *vps21Δ::hphMX4*	This study	Fig. 6B, E
YLY2156	YLY8442 *snf7Δ::hphMX4*	This study	Fig. 6B, E
YLY2157	YLY8442 *vps4Δ::hphMX4*	This study	Fig. 6B, E
YLY9023	YLY8442 *ypt7Δ::hphMX4*	[3]	Fig. 6B, E
YLY8470	SEY6210 *ATG8*::*mCherry-ATG8-TRP1 ATG11-3GFP::URA3*	[3]	Fig. 6C, E
YLY8668	YLY8470 *vps21Δ::LYS2*	[3]	Fig. 6C, E
YLY8590	YLY8470 *snf7Δ::hphMX4*	This study	Fig. 6C, E
YLY8589	YLY8470 *vps4Δ::hphMX4*	This study	Fig. 6C, E
YLY8610	YLY8470 *ypt7Δ::hphMX4*	[3]	Fig. 6C, E
YLY8471	SEY6210 *ATG8*::*mCherry-ATG8-TRP1 ATG17-3GFP::URA3*	[3]	Fig. 6D, E
YLY8611	YLY8471 *vps21Δ::LYS2*	[3]	Fig. 6D, E
YLY8593	YLY8471 *snf7Δ::hphMX4*	This study	Fig. 6D, E

YLY8592	YLY8471 *vps4Δ::hphMX4*	This study	Fig. 6D, E
YLY8669	YLY8471 *ypt7Δ::hphMX4*	[3]	Fig. 6D, E
YLY5239	YLY6880 *SNF7-mCherry::TRP1*	This study	Fig. 8A-C
YLY5241	YLY5239 *vps21Δ:: hphMX4*	This study	Fig. 8A-C
YLY1289	YLY2422 *SNF7-mCherry::TRP1*	This study	Fig. 8D, S5D; S3
YLY1282	YLY1289 *vps21Δ::hphMX4*	This study	Fig. 8D; Video 4
YLY1281	YLY8190 *VPS4-mNeonGreen::kanMX3*	This study	Fig. 8E-F, S5F
YLY1278	YLY1281 *vps21Δ:: hphMX4*	This study	Fig. 8E-F
YLY8190	SEY6210 *mCherry-ATG8::TRP1*	This study	Fig. 9A, S4, S5F
YLY4692	SEY6210 *ATG11-13Myc::TRP1 SNF7-3HA::HIS3*	This study	Fig. 9B
YLY10356	YLY4692 *vps21Δ::hphMX4*	This study	Fig. 9B
YLY10135	SEY6210 *ATG11-13Myc::TRP1 ATG17-3HA::HIS3*	This study	Fig. 9C
YLY10357	YLY10135 *vps21Δ::hphMX4*	This study	Fig. 9C
YLY10292	SEY6210 *SNF7-mCherry::TRP1 ATG17-3GFP::URA3*	This study	Fig. 10A
YLY10329	YLY10292 *vps21Δ::hphMX4*	This study	Fig. 10A
YLY7151	YLY10292 *ypt7Δ::hphMX4*	This study	Fig. 10A
YLY3671	SEY6210 *ATG17-3GFP::URA3*	This study	Fig. 10C-D
YLY3402	YLY3671 *vps21Δ::kanMX3*	This study	Fig. 10C-D
YLY3403	SEY6210 *SNF7::SNF7-GBP-mCherry*	This study	Fig. 10C-D
YLY3404	YLY3403 *vps21Δ::kanMX3*	This study	Fig. 10C-D
YLY3405	YLY3403 *ATG17-3GFP::URA3*	This study	Fig. 10C-D
YLY3406	YLY3404 *ATG17-3GFP::URA3*	This study	Fig. 10C-D
YLY915	SEY6210, *MATα ura3-52 leu2-3, 112 his3-Δ200 trp1-Δ901 lys2-801 suc2-Δ9 mel GAL*	[4]	Fig. 10D, S5C, S5E, S7A, S7C
YLY3401	SEY6210 *vps21Δ::kanMX3*	This study	Fig. 10D
YLY6449	YLY2422 *vps27Δ::kanMX3*	This study	Fig. S1, S2

YLY6450	YLY2422 *hse1Δ::kanMX3*	This study	Fig. S1, S2
YLY6583	YLY2422 *vps23Δ::kanMX3*	This study	Fig. S1, S2
YLY6446	YLY2422 *vps28Δ::kanMX3*	This study	Fig. S1, S2
YLY6447	YLY2422 *vps37Δ::kanMX3*	This study	Fig. S1, S2
YLY6448	YLY2422 *mvb12Δ::kanMX3*	This study	Fig. S1, S2
YLY6421	YLY2422 *vps22Δ::kanMX3*	This study	Fig. S1, S2
YLY6425	YLY2422 *vps36Δ::kanMX3*	This study	Fig. S1, S2
YLY6423	YLY2422 *vps25Δ::kanMX3*	This study	Fig. S1, S2
YLY6403	YLY2422 *vps20Δ::kanMX3*	This study	Fig. S1, S2
YLY6337	YLY2422 *vps24Δ::kanMX3*	This study	Fig. S1, S2
YLY6338	YLY2422 *vps2Δ::kanMX3*	This study	Fig. S1, S2
YLY6409	YLY2422 *vps60Δ::kanMX3*	This study	Fig. S1, S2
YLY6407	YLY2422 *did2Δ::kanMX3*	This study	Fig. S1, S2
YLY6427	YLY2422 *ist1Δ::kanMX3*	This study	Fig. S1, S2
YLY6336	YLY2422 *vta1Δ::kanMX3*	This study	Fig. S1, S2
YLY6451	YLY2422 *bro1Δ::kanMX3*	This study	Fig. S1, S2
YLY6452	YLY2422 *doa4Δ::kanMX3*	This study	Fig. S1, S2
YLY4752	YLY8190 *snf7Δ::hphMX4*	This study	Fig. S4
YLY4753	YLY8190 *vps4Δ::hphMX4*	This study	Fig. S4, S5F
YLY4757	YLY8190 *vps21Δ::hphMX4*	This study	Fig. S4A-B
YLY1026	YLY915, *SNF7-mCherry::TRP1*	This study	Fig. S5C
YLY7222	YLY915, *snf7Δ::hphMX4*	This study	Fig. S5C
YLY3830	YLY7222, *SNF7-mCherry::TRP1*	This study	Fig. S5C
YLY9953	YLY6405 *SNF7-mCherry::TRP1*	This study	Fig. S5D
YLY9265	YLY915 *vps4Δ::kanMX3*	This study	Fig. S5E

YLY9862	YLY915 *VPS4-mNeonGreen::kanMX3*	This study	Fig. S5E
YLY3086	YLY2422 *vps21Δ::LYS2*	[1]	Fig. S6
YLY6429	YLY3086 *vps4Δ::kanMX3*	This study	Fig. S6

B. Plasmids

Plasmid	Alias	Genotype	Source
pYL782	BFP-Pho8	pRS305-*pPGK1-3xtagBFP-PHO8*	[5]
pYL125	pRS423	2µ, *HIS3*, Amp	[6]
pYL662		pRS423-*VPS4*	This study
pYL664		pRS423-*SNF7*	This study
pYL1160	NeonGreen - Cps1	pRS416-*CPYp-NeonGreen-CPS1*	This study
pYL882	GFP	pFA6a-GFP-kanMX6	[7]
pYL1108	yEmCherry-Atg8	pRS304-yEmCherry-Atg8	[5]
pYL1252	Snf7-mCherry	ClhN-*SNF7-mCherry-TRP1*	This study
pYL1345		(pGEX-2T) *GST-VPS4*	[8]
pYL1346		(pGEX-2T) *GST -VPS4*^{K179A}	[8]
pYL1372	pGEX4T-1	2µ, Amp	Amersham Pharmacia Biotech
pYL1374		pGEX4T-1-*SNF7*	This study
pYL195	pYEX4T-1	*Cup, URA3, GST*	[9]
pYL1280		pYEX4T-1-*ATG17*	This study
pYL1277		pYEX4T-1-*SNF7*	This study
pYL272		*ATG17-3GFP-PG5(URA3)*	This study
pYL885		pUC119-*Padh81*-GBP-mCherry(C)-hphMX6-lys1*	[10]
pYL1437	Snf7-GBP-mCherry	pUC119-*Padh81-SNF7*-GBP-mCherry(C)-hphMX6-lys1*	This study
pYL502	pVC	pUG34-Venus-C (*HIS3*)	[11]
pYL1100		pVC-*SNF7*	This study
pYL1336		pVC-*SNF7(1-19, α0*)	This study
pYL1337		pVC-*SNF7(11-160, α1-α4*)	This study

pYL1338		pVC-*SNF7(140-240, MIM*)	This study
pYL1339		pVC-*SNF7(1-160, α0-α4*)	This study
pYL1340		pVC-*SNF7(11-240, α1-MIM*)	This study
pYL1341		pVC-*SNF7(*Δ*19-140,* Δ*α1-α4*)	This study
pYL501	pVN	pUG36-Venus-N (*URA3*)	[11]
pYL707		pVN-*ATG1*	This study
pYL726		pVN-*ATG11*	This study
pYL708		pVN-*ATG13*	This study
pYL760		pVN-*ATG17*	This study
pYL920		pVN-*ATG29*	This study
pYL1103		pVN-*ATG31*	This study

C. Oligos

Oligo name	Sequence
SNF7+500-forward	5'-CGCATCAAAGAAAGAGGTAG-3'
SNF7+500-reverse	5'-CAGGGCGAAGTAATCCAAAG-3'
SNF7+1000-forward	5'-CGTTGATTATTGGGTTTCTCC-3'
VPS4+500-forward	5'-GAGCGAGACAACCTCAAACC-3'
VPS4+500-reverse	5'-AGGAAAGCATCTCTGGGACT-3'
VPS4+1000-forward	5'-AGACACGCTTCACAAAGGAC-3'
ATG1+500-forward	5'-TTCTTTTTAACCGCTCGGCT-3'
ATG1+500-reverse	5'-GGATATGTATAGCCAAAGGC-3'
ATG1+1000-forward	5'-AAATAAGATACATTGCCGCTG-3'
VPS27+500-forward	5'-CGGAGCGACCATACACCATA-3'
VPS27+500-reverse	5'-TGGGAGGATTTACAAGACGA-3'
VPS27+1000-forward	5'-TGCTGCCAAACTAAACCAAGA-3'
HSE1+500-forward	5'-GCCCTCGGTCAAGAAGGATT-3'
HSE1+500-reverse	5'-CACGGTGGGTTTTAGGTTCA-3'
HSE1+1000-forward	5'-CACCAGTCAAGGCTTYCATCC-3'
VPS23+500-forward	5'-TCAAAATCACTGTACTTCTC-3'
VPS23+500-reverse	5'-GATGTTAAAGCCTCTGGCAG-3'
VPS23+1000-forward	5'-TGGCCCACATCAAAAACATC-3'
VPS28+500-forward	5'-ACGAGCAACAACAATAACAG-3'
VPS28+500-reverse	5'-ATCAGCAAAAGTAAAGATGG-3'
VPS28+1000-forward	5'-ACTTTGATGTGTCCACGCCGA-3'
VPS37+500 -forward	5'-TGCTTAACCGTCTTTTCTAT-3'

VPS37+500 -reverse	5'-AGTTCCATCTTTACCCCTCA-3'
VPS37+1000-forward	5'-GCCATTCTTATGTTGTTTCAG-3'
MVB12+500-forward	5'-ATGCTTTTGTGGATAGATAC-3'
MVB12+500-reverse	5'-TTCCTTGATAATGGTAGAGT-3'
MVB12+1000-forward	5'-GATACTGTCGTACTACCCAAG-3'
VPS22+500-forward	5'-CGGCTTGAATGTTACTGAAT-3'
VPS22+500-reverse	5'-TAGCACCTCGAATGCCCTTT-3'
VPS22+1000-forward	5'-TTTTGCTCCAAGGTAGTGCCA-3'
VPS36+500-forward	5'-AAGAGGGGGGAGTGGAGAG-3'
VPS36+500-reverse	5'-ACTGGTCATACGTGGCAGTT-3'
VPS36+1000-forward	5'-CGTCCTTCGATGATGTGTTTG-3'
VPS25+500-forward	5'-TTTTAGATATTTGCGTTAGC-3'
VPS25+500-reverse	5'-TGAGAATGAAGTCTTGGGAC-3'
VPS25+1000-forward	5'-TACTCCCAGTGTATTTGACCG-3'
VPS20+500-forward	5'-AATTCACAGCATCGTAGCCTT-3'
VPS20+500-reverse	5'-GTCATCTGCTTGGTTGGTTTC-3'
VPS20+1000-forward	5'-AATGGCAAGTTTTACGGATAG-3'
VPS24+500-forward	5'-CACTTCTTTCACTTCATCCAC-3'
VPS24+500-reverse	5'-CCTTTACGTTTCCGGCTTCTG-3'
VPS24+1000-forward	5'-AAGGTAAATCCGTCTGATGA-3'
VPS2+500 -forward	5'-AAGTGATATGCTCGAAAATG-3'
VPS2+500 -reverse	5'-AACTACATAAGGAGGCTGGT-3'
VPS2+1000-forward	5'-CGGTTGGTCTTATGTAGATA-3'
DID2+500-forward	5'-ACATAATGAGGAGGAGGTAA-3'
DID2+500-reverse	5'-GTAAGGATTTGGGATTTGAG-3'

DID2+1000-forward	5'-GGATGATTGTGACGGAGAGGA-3'
IST1+500-forward	5'-AGTGAATATGACACCAAGGG-3'
IST1+500-reverse	5'-TCGTTCAAGGCTCTGAGAAA-3'
IST1+1000-forward	5'-AACCTTGGTATGGAGGAGAGA-3'
VPS60+500-forward	5'-AAAAACTATTGGAGCAGGAGC-3'
VPS60+500-reverse	5'-GTTTACACAAATCGACACCGG-3'
VPS60+1000-forward	5'-GGAGGTGATCTTATTTGCGCT-3'
VTA1+500-forward	5'-ATCGGGGTTGGTCTCGTTAA-3'
VTA1+500-reverse	5'-ATTGCGGAACCATTGTCCTT-3'
VTA1+1000-forward	5'-TGATAGGTCCAGCGGTTCTG-3'
BRO1+500-forward	5'-ACCAGCAAGTACGAGAAGGA-3'
BRO1+500-reverse	5'-TTTAAGCGAAATCGAGGATG-3'
BRO1+1000-forward	5'-AAAACATCCCCTTCTCCTACA-3'
DOA4+500-forward	5'-ATGACGCTAGAATCGAACAA-3'
DOA4+500-reverse	5'-AAATCTAAAGGGGAGACGAA-3'
DOA4+1000-forward	5'-GCCTTGAATACTTGTTCCTAC-3'
VPS21+500-reverse	5'-TCGCCGATGAGTAGAAGATACAGAG-3'
VPS21+500-forward	5'-AAAGGTCTAATAAAAACGACTGGCG-3'
Ptef	5'-ACCCATGGTTGTTTATGTTC-3'
pRS423-Pst1-VPS4-forward	5'-GGGCTGAATCATGTTAGACACGCTTC-3'
VPS4-BamHI-pRS423-reverse	5'-GGGGGATCCGAATTGATAATGCTAGGGTA-3'
pRS423-Pst1- SNF7-forward	5'-GGGCTGCAGGGATCTAGGACCAGTAACAC-3'
SNF7-BamHI-pRS423-reverse	5'-GGGGGATCCCCATACACTAAAAACGGGTG-3'
pYEX4T-1-SmaI-SNF7-forward	5'-TCCCCCGGGATGTGGTCATCACTTTTTGG-3'

SNF7-SalI-pYEX4T-1-reverse	5'-GCGTCGACTCAAAGCCCCATTTCTGCTT-3'
pRS416-XhoI-CPYprmoter-forward	5'-GGGCTCGAGCTTCTGCACAAGAAGCCATATTGA-3'
CPYprmoter-NeonGreen-reverse	5'-CTTGCTCACCATAGCGTATGTATACTTTAAGTTGAGTAGAAAA-3'
CPYprmoter-NeonGreen-forward	5'-CATACGCTATGGTGAGCAAGGGCGAGG-3'
NeonGreen-EcoRI-pRS416-reverse	5'-GGGGAATTCCTTGTACAGCTCGTCCATGCC-3'
pRS416-BamHI-CPS1-forward	5'-GGGGGATCCATGATCGCCTTACCAGTAGA-3'
pRS416-CPS1-SacI-reverse	5'-GGGGAGCTCCCATATTCACTGCCTTTACG-3'
SNF7-pFA6a-forward	5'-GAAAAAGCATTAAGAGAACTACAAGCAGAAATGGGGCTTCGGATCCCCGGGTTAATTAA-3'
SNF7-pFA6a-reverse	5'-CCTTTTTTTTTCTTTCATCTAAACCGCATAGAACACGTGAATTCGAGCTCGTTTAAAC-3'
ATG17-pFA6a-forward	5'-TCTTCCCTGTACACTTTAAATTACAACGTGAAGAATCCTCGGATCCCCGGGTTAATTAA-3'
ATG17-pFA6a-reverse	5'-TTATTGAATCTTTGTACCGTATCCTTTTTTTCCTTTTTTGAATTCGAGCTCGTTTAAAC-3'
pYEX4T-1-SmaI-ATG17-forward	5'-TCCCCCGGGATGAACGAAGCAGATGTTAC-3'
ATG17-SalI-pYEX4T-1-reverse	5'-GCGTCGACCTAAGGATTCTTCACGTTGT-3'
pVC-SNF7(α0)-forward	5'-GGGGGATCCATGTGGTCATCACTTTTTGG-3'
pVC-SNF7(α1-α4)-forward	5'-GGGGGATCCAATGCCAAGAATAAAGAGTC-3'
pVC-SNF7(α1-α4)-reverse	5'-GGGGAATTCTCAATCCACCTCGTTTGCCCCAG-3'
pVC-SNF7(MIM)-forward	5'-GGGGGATCCGATGAAATAAGCGACGCTAT-3'
pVC-SNF7(MIM)-reverse	5'-GGGGAATTCTCAAAGCCCCATTTCTGCTT-3'
pVC-SNF7(a0-MIM)-forward	5'-AATGCCAAGAATAAAGAGTCAGATGAAATAAGCGACGCTAT-3'

add α0-forward	5'-GGGGGATCCATGTGGTCATCACTTTTTGGTTGGACATCAAGTAATGCCAAGAATAAAGA-3'
pVC-BamHI-SNF7-forward	5'-CGGGATCCATGTGGTCATCACTTTTTGG-3'
SNF7-EcoRI-pVC-reverse	5'-GGAATTCCATAGAACACGTTCAAAGCC-3'
pVN-BamHI-ATG31-forward	5'-CGGGATCCATGAATGTTACAGTTACTGT-3'
ATG31-EcoRI-pVN-reverse	5'-GGAATTCTCATACGGAATTGGAGAGCA-3'
pVN-XmaI-ATG29-forward	5'-CCCCCCGGGATGATTATGAATAGTACAAA-3'
ATG29-SalI-pVN-reverse	5'-GCGTCGACTTCAACCACTCTTGTAACCC-3'
pVN-SmaI-ATG13-forward	5'-CCCCCCGGGATGGTTGCCGAAGAGGACAT-3'
ATG13-SalI-pVN-reverse	5'-GCGTCGACTTAACCTTCTTTAGAAAGGT-3'
pVN-SmaI-ATG17-forward	5'-CCCCCCGGGATGAACGAAGCAGATGTTAC-3'
ATG17-SalI-pVN-reverse	5'-GCGTCGACCTAAGGATTCTTCACGTTGT-3'
pVN-SmaI-ATG1-forward	5'-CCCCCCGGGATGGGAGACATTAAAAATAA-3'
ATG1-SalI-pVN-reverse	5'-GCGTCGACTTAATTTTGGTGGTTCATCT-3'
pVN-SmaI-ATG11-forward	5'-CCCCCCGGGATGGCAGACGCTGATGAATA-3'
ATG11-SalI-pVN-reverse	5'-GCGTCGACTCAAACTCCCTGGTATGAAA-3'
HBKC-Ndel-SNF7-forward	5'-GGGCATATGATGTGGTCATCACTTTTTGG-3'
HBKC-BamH1-SNF7-reverse	5'-GGGGGATCCAAGCCCCATTTCTGCTTGTA-3'
VPS4-pFA6a-forward	5'-CAAGAACAGTTCACTAGAGATTTTGGTCAAGAAGGTAACCGGATCCCCGGGTTAATTAA-3'
VPS4-pFA6a-reverse	5'-TGTACACAAGAAATCTACATTAGCACGTTAATCAATTGAGAATTCGAGCTCGTTTAAAC-3'

References

1. Chen, Y., et al., *A Vps21 endocytic module regulates autophagy.* Mol Biol Cell, 2014. **25**(20): p. 3166-77.
2. Noda, T., et al., *Novel system for monitoring autophagy in the yeast Saccharomyces cerevisiae.* Biochem Biophys Res Commun, 1995. **210**(1): p. 126-32.
3. Zhou, F., et al., *A Rab5 GTPase module is important for autophagosome closure.* PLoS Genet, 2017. **13**(9): p. e1007020.
4. Robinson, J.S., et al., *Protein sorting in Saccharomyces cerevisiae: isolation of mutants defective in the delivery and processing of multiple vacuolar hydrolases.* Mol Cell Biol, 1988. **8**(11): p. 4936-48.
5. Graef, M., et al., *ER exit sites are physical and functional core autophagosome biogenesis components.* Mol Biol Cell, 2013. **24**(18): p. 2918-31.
6. Sikorski, R.S. and P. Hieter, *A system of shuttle vectors and yeast host strains designed for efficient manipulation of DNA in Saccharomyces cerevisiae.* Genetics, 1989. **122**(1): p. 19-27.
7. Longtine, M.S., et al., *Additional modules for versatile and economical PCR-based gene deletion and modification in Saccharomyces cerevisiae.* Yeast, 1998. **14**(10): p. 953-61.
8. Babst, M., et al., *Endosomal transport function in yeast requires a novel AAA-type ATPase, Vps4p.* EMBO J, 1997. **16**(8): p. 1820-31.
9. Morozova, N., et al., *TRAPPII subunits are required for the specificity switch of a Ypt-Rab GEF.* Nat Cell Biol, 2006. **8**(11): p. 1263-9.
10. Chen, Y.H., et al., *Facile manipulation of protein localization in fission yeast through binding of GFP-binding protein to GFP.* J Cell Sci, 2017. **130**(5): p. 1003-1015.
11. Gong, T., et al., *Control of Polarized Growth by the Rho Family GTPase Rho4 in Budding Yeast: Requirement of the N-Terminal Extension of Rho4 and Regulation by the Rho GTPase-Activating Protein Bem2.* Eukaryotic Cell, 2013. **12**(2): p. 368-377.
